# *Moraxella catarrhalis* Restriction–Modification Systems Are Associated with Phylogenetic Lineage and Disease

**DOI:** 10.1093/gbe/evy226

**Published:** 2018-10-18

**Authors:** Luke V Blakeway, Aimee Tan, Rachael Lappan, Amir Ariff, Janessa L Pickering, Christopher S Peacock, Christopher C Blyth, Charlene M Kahler, Barbara J Chang, Deborah Lehmann, Lea-Ann S Kirkham, Timothy F Murphy, Michael P Jennings, Lauren O Bakaletz, John M Atack, Ian R Peak, Kate L Seib

**Affiliations:** 1 Institute for Glycomics, Griffith University, Gold Coast, Queensland, Australia; 2 The Marshall Centre for Infectious Diseases Research and Training, School of Biomedical Sciences, The University of Western Australia, Perth, Western Australia, Australia; 3 Wesfarmers Centre of Vaccines and Infectious Diseases, Telethon Kids Institute, The University of Western Australia, Perth, Western Australia, Australia; 4 School of Biomedical Sciences, The University of Western Australia, Perth, Western Australia, Australia; 5 School of Medicine, The University of Western Australia, Perth, Western Australia, Australia; 6 Department of Infectious Diseases, Perth Chilren's Hospital, Perth, Western Australia, Australia; 7 Department of Microbiology, PathWest Laboratory Medicine, QEII Medical Centre, Perth, Western Australia, Australia; 8 Clinical and Translational Research Center, University at Buffalo, the State University of New York, Buffalo, New York, USA; 9 Center for Microbial Pathogenesis, The Research Institute at Nationwide Children’s Hospital, Columbus, Ohio, USA; 10 Department of Pediatrics, The Ohio State University College of Medicine, Columbus, Ohio, USA; 11 School of Medical Science, Griffith University, Gold Coast, Queensland, Australia

**Keywords:** *Moraxella catarrhalis*, restriction–modification systems, allelic variation, speciation, genome analysis, otitis media

## Abstract

*Moraxella catarrhalis* is a human-adapted pathogen, and a major cause of otitis media (OM) and exacerbations of chronic obstructive pulmonary disease. The species is comprised of two main phylogenetic lineages, RB1 and RB2/3. Restriction–modification (R-M) systems are among the few lineage-associated genes identified in other bacterial genera and have multiple functions including defense against foreign invading DNA, maintenance of speciation, and epigenetic regulation of gene expression. Here, we define the repertoire of R-M systems in 51 publicly available *M. catarrhalis* genomes and report their distribution among *M. catarrhalis* phylogenetic lineages. An association with phylogenetic lineage (RB1 or RB2/3) was observed for six R-M systems, which may contribute to the evolution of the lineages by restricting DNA transformation. In addition, we observed a relationship between a mutually exclusive Type I R-M system and a Type III R-M system at a single locus conserved throughout a geographically and clinically diverse set of *M. catarrhalis* isolates. The Type III R-M system at this locus contains the phase-variable Type III DNA methyltransferase, *modM*, which controls a phasevarion (phase-variable regulon). We observed an association between *modM* presence and OM-associated middle ear isolates, indicating a potential role for ModM-mediated epigenetic regulation in OM pathobiology.

## Introduction

*Moraxella catarrhalis* is a Gram-negative bacterial colonizer of the human respiratory tract. Although often carried asymptomatically in the nasopharynx (Vaneechoutte et al. 1990), acquisition of *M. catarrhalis* frequently progresses to otitis media (OM) in infants and children, and exacerbations of chronic obstructive pulmonary disease (COPD) in adults. It is one of the three most prevalent bacterial causes of OM, along with *Streptococcus pneumoniae* and nontypeable *Haemophilus influenzae* (NTHi), and is detected in middle ear fluid in up to 56% of OM cases by PCR (Pettigrew et al. 2017). *Moraxellacatarrhalis* is the second most prevalent pathogen associated with exacerbations of COPD, after NTHi (Sethi and Murphy 2008; Murphy and Parameswaran 2009), and accounts for ∼10% of exacerbations in the USA per year (Murphy et al. 2005). The progression from asymptomatic carriage of *M. catarrhalis* to symptomatic disease is poorly understood but likely involves both host and bacterial factors (Gisselsson-Solen et al. 2014). There is currently no available vaccine to prevent *M. catarrhalis*-mediated disease.

The *M. catarrhalis* species is a panmictic population of strains (Enright and McKenzie 1997; Wirth et al. 2007) that is divided into two main phylogenetic lineages based on molecular typing methods such as multilocus sequence typing (MLST) (Wirth et al. 2007), pulsed field gel electrophoresis (PFGE) (Verhaegh et al. 2011), and single-adapter amplified fragment length polymorphism (sAFLP) (Bootsma et al. 2000). The major lineage includes ∼80–90% of isolates and is primarily comprised of 16S ribotype 1 (RB1) strains, whereas the remaining ∼10–20% of isolates belong to the minor lineage and consist of 16S ribotype 2 and 3 (RB2/3) strains (Bootsma et al. 2000; Verhaegh et al. 2008, 2011). There is some evidence to suggest that propensity to cause disease is correlated with phylogenetic lineage. For example, 51% of RB1 lineage isolates were associated with disease cases compared with 14% of RB2/3 strains in one study (Wirth et al. 2007), and RB1 lineage strains exhibit higher levels of adherence to airway epithelial cells, and increased resistance to killing by human serum in vitro than RB2/3 lineage strains, with some exceptions (Bootsma et al. 2000; Wirth et al. 2007; Earl et al. 2016). However, a clear genetic basis for the proposed difference in virulence between *M. catarrhalis* phylogenetic lineages has not been established. *Moraxellacatarrhalis* expresses a repertoire of outer membrane proteins involved in adherence to epithelial cells and the extracellular matrix, serum resistance, and iron acquisition that are considered to be major virulence factors (Blakeway et al. 2017). Comparative supragenome analysis of thirteen RB1 and eighteen RB2/3 genomes demonstrated that virulence factors responsible for adherence and serum resistance are found in the core *M. catarrhalis* genome, with no virulence factors found solely in either lineage (Earl et al. 2016). This is supported by a recent review of the distribution of virulence factors, which suggests that lineage-associated allelic variation and expression differences potentially underpin the differential pathogenicity among *M. catarrhalis* isolates (Blakeway et al. 2017).

In many bacterial species, restriction–modification (R-M) systems are among the few genes associated with distinct phylogenetic lineages or virulent versus avirulent subpopulations (Budroni et al. 2011; Seib et al. 2011; Roberts et al. 2013; Nandi et al. 2015; Tan, Hill, et al. 2016). R-M systems are ubiquitous in bacteria and are particularly abundant in naturally competent species, for example, *Neisseria meningitidis* (Budroni et al. 2011; Kong et al. 2013), *Neisseria gonorrhoeae* (Stein et al. 1995), *H. influenzae* (Vasu and Nagaraja 2013), *S. pneumoniae* (Vasu and Nagaraja 2013), and *Helicobacter pylori* (Lin et al. 2001; Kumar et al. 2015). R-M systems consist of a restriction endonuclease and a DNA (adenine or cytosine) methyltransferase that cleave and methylate DNA at specific DNA sequences, respectively. R-M systems have traditionally been described as a type of bacterial defense mechanism to protect the host cell from invasive foreign DNA (e.g., bacteriophages) (Bickle and Kruger 1993). However, R-M systems have been demonstrated to perform several additional roles, including maintaining speciation (Vasu and Nagaraja 2013), DNA repair (Vasu and Nagaraja 2013), and epigenetic regulation of gene expression (Srikhanta et al. 2005), reviewed in Atack et al. (2018). Four main types of R-M systems exist (Types I–IV) that differ in their subunit composition, cofactor requirements and mechanism of action (Roberts et al. 2003). Type I R-M systems are a complex of three subunits: A restriction endonuclease (HsdR) that cleaves unmethylated DNA, a DNA methyltransferase (HsdM) that methylates DNA and protects the host genome from cleavage, and a specificity subunit (HsdS) that determines the recognition sequence of the complex. Type II R-M systems consist of a restriction endonuclease (Res) and a methyltransferase (Mod) that act independently of each other. Type III R-M systems consist of an independent methyltransferase (Mod) that contains a DNA target recognition domain (TRD; also known as the DNA recognition domain) and methylates DNA, and a restriction endonuclease (Res) that must form a complex with Mod to recognize and cleave DNA. Type IV R-M systems are composed of a single enzyme that only cleaves methylated DNA. Many host-adapted bacterial pathogens contain R-M systems that are phase-variable. Phase variation is the random and reversible, high frequency on/off or graded switching of gene expression, which is typically mediated by simple DNA repeats in host adapted bacteria. However, various mechanisms mediate phase variation, including slipped strand mispairing of simple DNA sequence repeats (van Ham et al. 1993), site-specific recombination (e.g., DNA inversion, Zieg et al. 1977 or domain shuffling, Manso et al. 2014) or epigenetic mechanisms (e.g., differential methylation and competition between regulatory proteins Dam and Lrp affect expression of the *Escherichia coli pap* operon; van der Woude et al. 1996). Phase variation of DNA methyltransferases due to changes in DNA sequence repeats (Srikhanta et al. 2005, 2009; Seib et al. 2011; Blakeway et al. 2014) or domain shuffling (Manso et al. 2014), causes differential methylation of the genome, which epigenetically alters the expression of multiple genes in systems known as phasevarions (phase-variable regulons) (Srikhanta et al. 2005; Atack et al. 2018). In every case identified, switching of expression of phasevarions alters the pathobiology of the organism, and controls expression of current and putative vaccine candidates (Atack et al. 2018).

To date only four R-M systems have been described in detail in *M. catarrhalis*: Mca25239IIP (Type I) (Blakeway et al. 2014); Mca25239ORF17P (Type II) (Blakeway et al. 2014); Mca25239IP (Type III) (Seib et al. 2002; Blakeway et al. 2014); and Mca23246IIP (Type III) (Seib et al. 2002). We previously identified that the Type III DNA methyltransferases ModM (Mca25239Imod) and ModN (Mca23246IIPmod) are phase variable, and that there are three *modM* alleles (*modM1-3*) that vary in their TRDs (Seib et al. 2002; Blakeway et al. 2014). We have also shown that the phase-variable Type III methyltransferase ModM2 controls expression of multiple genes in a phasevarion, including genes involved in colonization, and protection against host defenses (Blakeway et al. 2014). For example, genes differentially expressed between *modM2* ON and *modM2* OFF variants are involved in infection in the chinchilla model (e.g., MiaE, FbpA; Hoopman et al. 2012); attachment to pharyngeal or alveolar cells (e.g., RpmG, AhcY; de Vries et al. 2013); and oxidative stress responses (e.g., FbpA, BfrA; Hoopman et al. 2011). We also reported the distribution of the phase variable *modM* alleles M.Mca25239I (*modM2*) and M.Mca195I (*modM3*) in a panel of middle ear and nasopharyngeal carriage isolates, and observed a significant association of the *modM3* allele with middle ear isolates from patients with OM (Blakeway et al. 2014). However, the full repertoire and distribution of R-M systems in the *M. catarrhalis* has never before been described. In this study we examine the distribution of R-M systems identified in 51 publicly available *M. catarrhalis* genomes. We also characterize the variable R-M system loci found in *M. catarrhalis*, and specifically investigate the distribution of the phase-variable *modM* locus in geographically and clinically diverse panels of *M. catarrhalis* isolates.

## Materials and Methods

### Characterization of *M. catarrhalis* R-M Systems

The nucleotide sequences of all predicted R-M systems in five closed *M. catarrhalis* genomes (BBH18, de Vries et al. 2010, 25239, Blakeway et al. 2014, 25240, Daligault et al. 2014, FDAARGOS_213 [Accession NZ_CP020400], and CCRI-195ME, Tan et al. 2017) listed in the REBASE database (Roberts et al. 2010) were acquired from GenBank (Benson et al. 2017). Additional R-M system loci were identified through a search for annotated R-M systems in draft *M. catarrhalis* genomes (e.g., the novel R-M system McaC031IP), and by literature search (e.g., R-M system Mca23246IIP has been previously described, Seib et al. 2002). Multiple sequence alignments of 10–20 kb regions upstream and downstream of each R-M system were then performed between the five closed genome strains in order to identify conserved flanking genes. Each of the identified R-M systems, along with its conserved flanking genes, were defined as a distinct R-M system locus and assigned a locus number. Two putative phage encoded DNA methyltransferases, M.Mca195ORF1235P and M.Mca195ORF2720P, were found but not included in phylogenetic analysis as their presence/absence could not be accurately determined in all genomes. A representative of each R-M system is shown in table 1. A custom BLAST database was then created containing 51 publicly available *M. catarrhalis* genomes (5 closed genomes and 46 draft genomes) using Geneious version 10.1.3 (http://www.geneious.com; last accessed October 23, 2018; Kearse et al. 2012). The genome 157.rep2_MCAT was excluded from the database as it is a resequence of 157.rep1_MCAT and not a unique *M. catarrhalis* isolate.

**Table 1:** Representative R-M Systems at Each Locus in *Moraxella catarrhalis* Strains

Locus	Name	Predicted Type	Subunits	Av. GC	Accession	Coordinates
1	Mca25240ORF1547P	I	M, R, S1, S2	39.3%	NZ_CP008804.1	1,653,709–1,663,848
	Mca25239IP (*modM*)	III	M, R	33.1%	NZ_CP007669.1	427,245–431,803
2	Mca25239ORF528P	IIM	R	30.4%	NZ_CP007669.1	591,870–593,629
	Mca7169IP	IIS	M1, M2, R	31.5%	AERC01000019.1	160,541–163,092
	McaA9IP	IIG	R/M	35.5%	NZ_LXHW01000022.1	82,890–84,937
	McaZ7547IP	IV	R	30.0%	LXHD01000025.1	50,224–51,273
3	McaCO72IP	II	M, R	35.5%	AERK01000012.1	53,470–55,328
4	Mca25239MrrP	IV	R	36.6%	NZ_CP007669.1	1,203,209–1,204,152
5	Mca25239ORF1708P^a^	III	M, R	36.9%	NZ_CP007669.1	1,831,862–1,836,731
	McaBBH18IP	II	M, R	30.8%	NC_014147.1	1,763,372–1,765,715
	Mca213ORF2330P	II	M, R	31.1%	NZ_CP020400.1	473,956–475,872
6	Mca25239ORF1739P	I	S	36.0%	NZ_CP007669.1	1,866,245–1,866,910
7	Mca25239ORF17P	II	M, R	29.9%	NZ_CP007669.1	18,453–20,511
	McaS11IP	II	M, R	34.3%	LXHF01000024.1	8,509–10,532
	McaF21IP	II	M	34.0%	LXHR01000010.1	9,304–10,161
8	Mca25239IIP	I	M, R, S	41.4%	NZ_CP007669.1	79,387–87,869
9	Mca195ORF6035P	II	M, R	32.5%	NZ_CP018059.1	1,237,296–1,238,994
10	McaC031IP (*modO*)	III	M, R	39.9%	LXHV01000037.1	80,716–85,836
11	Mca23246IIP (*modN*)	III	M, R	36.5%	AY049057	371–3,295
12	Mca25239ORF1708P^a^	III	M, R	38.5%	NZ_CP007669.1	1,831,862–1,836,731

Note.—M, modification enzyme; R, restriction endonuclease; S, specificity subunit; R/M, fused restriction endonuclease and modification domains.

a Same R-M system present at locus 5 and locus 12.

**Fig. 1.**
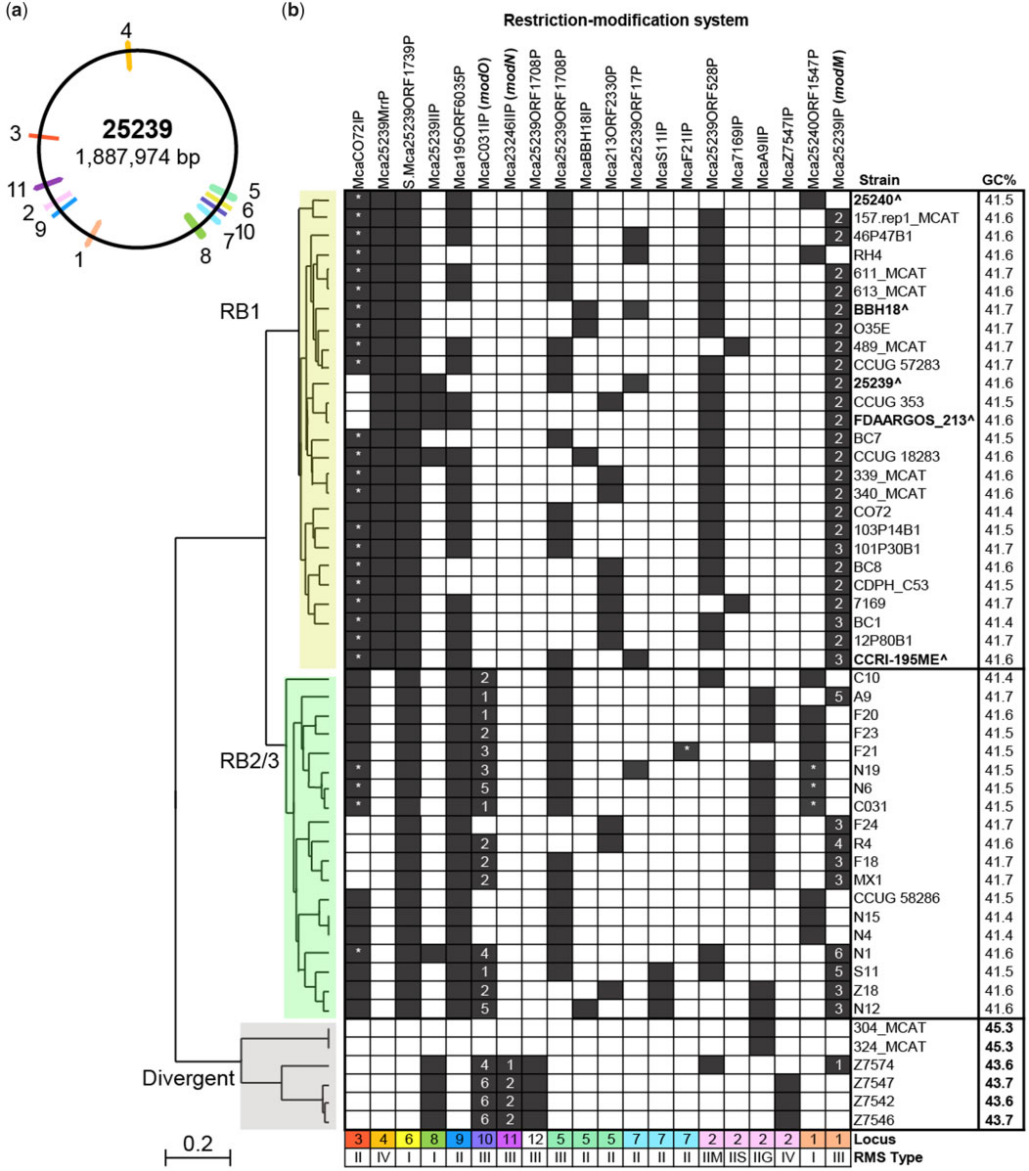
—Distribution of restriction–modification (R-M) systems in *Moraxella catarrhalis* genomes. (*a*) The positions of R-M system loci mapped to the *M. catarrhalis* strain 25239 chromosome. R-M system loci positions are conserved in the five closed genome strains relative to each other. Locus 12 is unable to be mapped due to chromosomal rearrangement. (*b***)** Presence/absence matrix of 18 putative R-M systems and an orphan Type I R-M system specificity subunit (locus 6) in 51 publicly available *M. catarrhalis* genomes. Each row represents one *M. catarrhalis* genome and each column represents one R-M system. Black/white squares indicate the presence or absence of the R-M system in the genome, respectively. ^ and bold font indicates the five *M. catarrhalis* closed genome strains. Numbers 1–6 indicate Type III R-M system DNA methyltransferase alleles for *modM* (locus 1), *modN* (locus 11), and *modO* (locus 10). * Indicates R-M systems where the restriction endonuclease gene is absent. Phylogenetic tree: Yellow = RB1 lineage, green = RB2/3 lineage, and grey = divergent phylogenetic lineages. Phylogenetic analysis has not been performed for strains 12P80B1 and CCRI-195ME by NCBI, however both are 16S ribotype RB1 and are grouped with RB1 lineage strains.

Novel R-M systems at the defined R-M system loci were identified by using the conserved flanking genes at each R-M system locus as the query sequences in a BlastN search against all 51 genomes and aligning the region between the flanking sequences. A sequence was defined as a putative R-M system when at least one R-M system gene (methyltransferase, restriction endonuclease, or specificity subunit) was present, regardless of missing subunits or inactivating mutations. R-M systems were considered to be the same system if >70% nucleotide sequence identity to the reference strain was observed in the DNA methyltransferase and/or restriction endonuclease genes, when present. Each predicted *M. catarrhalis* R-M system was queried against the REBASE database using BlastN and named according to the best REBASE hit or accepted nomenclature (Roberts et al. 2003) in the case of previously undescribed, putative R-M systems. Sequences of all putative R-M systems were then used in a BlastN search against all 51 genomes to determine if any of the identified R-M systems were found at additional loci that were not previously defined. A presence/absence matrix of R-M systems in 51 *M. catarrhalis* genome strains was then constructed (fig. 1).

### Sequence Alignments

General sequence alignments and pairwise identity calculations were performed using ClustalW and Geneious version 10.1.3 (http://www.geneious.com; last accessed October 23, 2018) (Kearse et al. 2012), with the following parameters: Cost matrix = IUB, gap open cost = 15, gap extend cost = 6.66. Alignments and visualization of multiple R-M systems were performed using Easyfig version 2.2.2 (Sullivan et al. 2011). Alignments of *modM* alleles were performed using ClustalW as above and visualized with JalView (http://www.jalview.org/; last accessed October 23, 2018).

### Phylogenetic Analysis

The *M. catarrhalis* (species ID: 1232) phylogenetic dendrogram was sourced from NCBI GenBank (Benson et al. 2017), accessed at https://www.ncbi.nlm.nih.gov/genome/? term=moraxella+catarrhalis; last accessed October 23, 2018. The *Moraxella* genus phylogenetic tree was drawn with MEGA7 (Kumar et al. 2016) by applying the maximum likelihood method with Kimura-2 genetic distance model and 1,000 bootstrap replicates to 16S rRNA sequences obtained from the All-Species Living Tree Project database (Yarza et al. 2008). Homologs of *M. catarrhalis* Type I R-M system specificity subunit TRDs and Type III R-M system DNA methyltransferase TRDs in other species were identified by BlastX search against the NCBI nr database, excluding hits from *M. catarrhalis*.

### R-M System Screening by Multiplex PCR

To identify the R-M system present at locus 1 in a large panel of unsequenced strains, multiplex PCR reactions were performed. Panels of *M. catarrhalis* strains used in this study are detailed in table 2. *Moraxellacatarrhalis* strains were grown on brain heart infusion (BHI) agar (Oxoid, Basingstoke, UK) at 37 °C with 5% CO_2_. Multiplex PCR reactions were performed using GoTaq Flexi DNA polymerase (Promega, Wisconsin, USA) as per the manufacturer’s instructions (55 °C annealing, 60 s elongation, 30× cycles, 10 µmol each primer, 25 µl reaction volume) with 1 ng genomic DNA or 1 µl of boiled cell lysate as template. The eight multiplex PCR primers used are detailed in supplementary table S1, Supplementary Material online. Amplicons were resolved by electrophoresis on a 1.5% agarose gel. A different sized amplicon was generated depending on the R-M system present. Type I R-M system (Mca25240ORF1547P): 600 bp, *modM1* (M.Mca23246IP): 400 bp, *modM2* (M.Mca25239I): 300 bp, *modM3* (M.Mca195I): 500 bp, (supplementary table S1 and fig. S1, Supplementary Material online).

**Table 2:** Panels of *Moraxella catarrhalis* Strains

			Child		Adult		
Panel (Ref)	Location	Date	ME^^^	NP	Sputum	Clinical	Total
BRPRU (Blakeway et al. 2014)	Columbus	2004–2010	17	108	—	—	**125**
	Ohio, USA						
KOMRP (Lehmann et al. 2008)	Kalgoorlie-Boulder	1999–2004	—	103	—	—	**103**
	WA, Australia						
GROMIT (Wiertsema et al. 2011)	Perth	2007–2009	7	120	—	—	**127**
	WA, Australia						
biOMe (Lappan et al. 2018)	Perth,	2013–2015	8	47	—	—	**55**
	WA, Australia						
PathWest (Ariff et al. 2015)	Perth	2003–2012	—	—	—	41	**41**
	WA, Australia						
USA COPD (Murphy et al. 2005)	Buffalo	1994–2000	—	—	98	—	**98**
	New York, USA						
		**Total**	**32**	**378**	**98**	**41**	**549**

Note.—ME, isolated from middle ear fluid at the time of otitis media; NP, isolated from the nasopharynx of children with a history of OM or healthy control children; Sputum, isolated from sputum of COPD patients during periods of exacerbation or stable colonization; Clinical, carriage or disease association not specified.

### Statistical Analysis

*P*-values were determined by a two-tailed Fisher’s exact test using Graphpad Prism Version 6.01. The significance threshold was set at 0.05.

## Results

### Distribution of *M. catarrhalis* R-M Systems in 51 Genomes

**Fig. 2.**
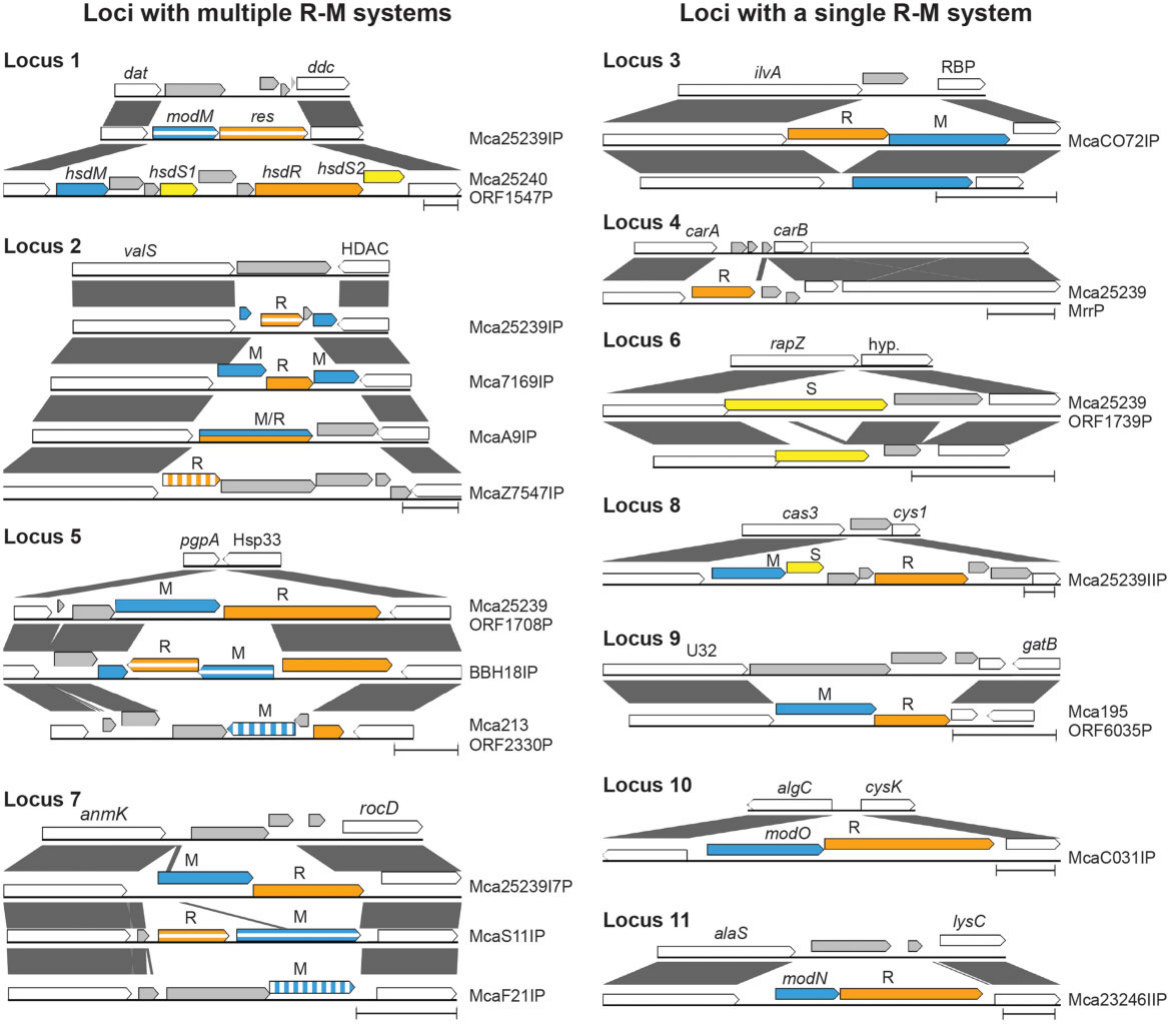
—Comparison of sequence variants at *Moraxella catarrhalis* R-M system loci. A pairwise BlastN comparison is shown for each R-M system variant present at loci 1–11. Colored arrows represent genes present within R-M system loci: White = conserved flanking gene (gene name given above arrow), blue = methyltransferase (M), orange = restriction endonuclease (R), yellow = specificity subunit (S), grey = genes that do not encode R-M system subunits. Different R-M systems present in the same locus are variously indicated by solid, vertical striped, or horizontal striped arrows. Nucleotide sequence identity across a run of nucleotides between sequence variants at a particular R-M system loci is indicated by grey lines. Scale bars represent 1 kb.

In order to determine the distribution and variability of R-M systems in *M. catarrhalis*, all loci harboring predicted R-M systems were investigated in five closed and 46 draft *M. catarrhalis* genomes. Of the 51 total genomes analyzed, 26 are RB1 lineage strains, 19 are RB2/3 lineage strains, and six are divergent strains that differ in their average GC content (43.6–45.3%) relative to RB1 and RB2/3 lineage strains (41.6%) (fig. 1), and although currently classified as *M. catarrhalis*, are predicted to represent a different species or subspecies (Wirth et al. 2007; Earl et al. 2016). Nineteen putative R-M systems were identified in this study at twelve distinct genetic loci (loci 1–12) (table 1, figs. 1 and 2). Seven loci (loci 3, 4, 6, 8, 9, 10, & 11) each contain a unique R-M system that is found at only a single locus (figs. 1 and 2). Locus 12 contains a single R-M system (Mca25239ORF1708P), however this R-M system is not unique to locus 12 and is also found at locus 5. Four loci (loci 1, 2, 5, & 7) are variable, each containing two to four mutually exclusive R-M systems distributed between different strains (figs. 1 and 2). All strains possessed between one (e.g., strain 304_MCAT) and eight (e.g., strain 46P47B1) putative R-M systems (fig. 1). None of the genomes analyzed contained all 12 R-M systems, and every R-M system except for McaF21IP was found in at least two genomes.

### *Moraxella catarrhalis* R-M Systems Are Associated with Phylogenetic Lineage

A presence/absence matrix of R-M systems in 51 *M. catarrhalis* strains was generated, with strains ordered based on phylogeny (fig. 1). Comparison of R-M systems between the 26 RB1 lineage strains and 19 RB2/3 lineage strains found that the presence of R-M systems at five loci (loci 1, 2, 3, 4, 10) correlate with phylogenetic lineage. Locus 1 contains either a Type I R-M system (Mca25240ORF1547P) or a Type III R-M system (Mca25239IP) (figs. 1 and 2, table 1). The Type III R-M system is associated with the RB1 lineage (present in 24/26 RB1 vs 9/19 RB2/3 strains; *P* = 0.0014) whereas the mutually exclusive Type I R-M system is associated with the RB2/3 lineage (present in 2/26 RB1 vs 10/19 RB2/3 strains; *P* = 0.0014). At locus 2, four putative Type II R-M systems (Mca25239ORF528P, McaA9IIP, Mca7169IP, or Mca7547IP) are found that are mutually exclusive and differentially distributed between strains (fig. 2); Mca25239ORF528P is associated with the RB1 lineage (present in 22/26 RB1 vs 3/19 RB2/3 strains; *P* < 0.0001), whereas McaA9IIP is associated with the RB2/3 lineage (present in 0/26 RB1 vs 12/19 RB2/3 strains; *P* < 0.0001). Locus 3 contains a putative Type II R-M system McaCO72IP (fig. 2). Although no significant difference was observed in the distribution of the methyltransferase gene (M.McaCO72IP) between lineages (present in 23/26 RB1 vs 15/19 RB2/3; *P* = 0.4329), the associated restriction endonuclease gene (R.McaCO72IP) is only present in one RB1 strain (CO72) compared with 11/19 RB2/3 strains (*P* < 0.0001). The putative Type IV R-M system Mca25239MrrP at locus 4 is present in all 26 RB1 lineage strains, whereas it is absent from all 19 RB2/3 strains (*P* < 0.0001) and three short open reading frames (ORFs) encoding hypothetical proteins are present instead (fig. 2). Conversely, the predicted Type III R-M system McaC031IP at locus 10 (fig. 2) is associated with the RB2/3 lineage (present in 0/26 RB1 vs 15/19 RB2/3 lineage strains; *P* < 0.0001).

Differences in the presence of R-M systems are also observed between *M. catarrhalis* RB1 and RB2/3 strains compared with strains of the divergent group. Two R-M systems, McaZ7547IP at locus 2 and Mca23246IIP at locus 11 (fig. 2), are associated with the divergent group (each present in 4/6 divergent vs 0/45 RB1 and RB2/3 strains; *P* < 0.00001). Conversely, two putative Type II R-M systems, McaCO72IP at locus 3 (fig. 2) and Mca195ORF6035P at locus 9 (fig. 2), are associated with RB1 and RB2/3 strains (each present in 0/6 divergent vs 38/45 RB1 and RB2/3 strains; *P* < 0.00001). The putative Type III R-M system Mca25239ORF1708P was present in strains from all lineages, however it was found at locus 5 in RB1 and RB2/3 strains but at locus 12 in divergent strains. Differences in R-M system presence were also observed between strains within the divergent group. Five to six R-M systems are present in strains Z7542, Z7546, Z7547, and Z7574, whereas strains 304_MCAT and 324_MCAT harbored only one R-M system, McaA9IIP at locus 2 (fig. 2), which is absent in the aforementioned divergent strains.

### Variability within Type I R-M Systems

**Fig. 3.**
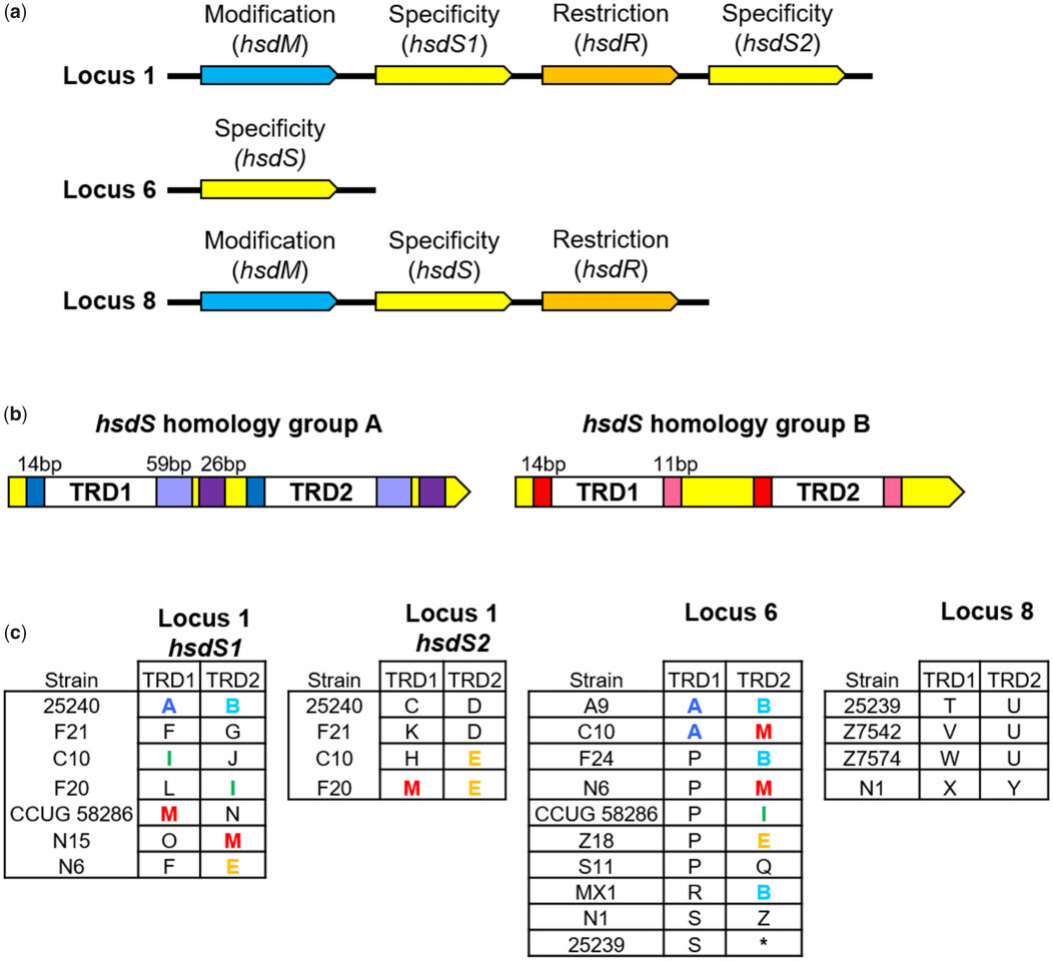
—Schematic of variable *hsdS* target recognition domains (TRDs) of Type I DNA methyltransferases. (*a*) Schematic representation Type I R-M systems at loci 1, 6, and 8, indicating the arrangement of their modification, specificity and restriction endonuclease subunit genes. (*b*) Sequence organization of *hsdS* genes belonging to homology group A and homology group B. Colored boxes adjacent to the two variable TRDs (TRD1 and TRD2) indicate repeated sequences within a *hsdS* gene, at which homologous recombination may occur, mediating domain movement of TRDs. (*c*) Variable TRDs present within the *hsdS* genes of the complete Type I R-M systems (locus 1 and locus 8) and the orphan *hsdS* gene (locus 6) are shown. Each of the 26 unique TRDs are assigned a letter (A–Z) and representative strains possessing each TRD are shown. Colored letters indicate TRDs that have undergone domain movement and are found at more than one locus. *No TRD is present due to a truncated *hsdS* gene.

Type I R-M systems are a complex of three subunits: Restriction endonuclease (HsdR), DNA methyltransferase (HsdM), and a specificity subunit (HsdS) that is comprised of two TRDs that recognize bipartite sequences and together determine the recognition sequence of the Type I R-M system complex (Murray 2000). Three putative Type I R-M systems were found in the 51 genomes analyzed, at loci 1, 6 and 8 (fig. 1, table 1). Locus 1 (Mca25240ORF1547P) and locus 8 (Mca25239IIP) harbor complete Type I R-M systems, comprised of proximally located *hsdM, hsdR*, and *hsdS* genes; however, locus 6 contains an orphan *hsdS* gene (S.Mca25239ORF1739P) with no proximal *hsdR* or *hsdM* genes identified (fig. 3*a*). Interestingly, Mca25240ORF1547P (locus 1) has two *hsdS* genes (S1.Mca25240ORF1547P; “*hsdS1*,” and S2.Mca25240ORF1547P; “*hsdS2*”) (fig. 3*a*), in all strains except for C031, N6, and N19, in which the *hsdS2* and *hsdR* genes (R.Mca25240ORF1547P) are absent (fig. 1). Separate nucleotide sequence alignments of locus 1 and locus 8 R-M systems found that *hsdR* and *hsdM* genes were well conserved within their respective loci (88–99% nucleotide identity); however, when comparing *hsdR* and *hsdM* genes between loci 1 and 8 only 44% nucleotide identity was observed, suggesting that these are distinct R-M systems at loci 1 and 8.

Nucleotide sequences of all four *hsdS* genes across the three loci were aligned and although the TRDs were highly variable, two *hsdS* homology groups were identified based on the presence of homologous regions that are unique to each homology group, adjacent to the variable TRDs (fig. 3*b*). The *hsdS* homology group A consists of the locus 1 *hsdS1*, locus 1 *hsdS2*, and locus 6 orphan *hsdS* gene, while homology group B contains only the locus 8 *hsdS* gene. The homologous regions consisted of directly repeated sequences between 11 and 59 base pairs occurring upstream and downstream of both TRDs within a *hsdS* gene, potentially permitting recombination between TRDs. Twenty unique TRDs were identified in 21 combinations of *hsdS* genes belonging to homology group A and six TRDs in four *hsdS* genes were identified in homology group B. Evidence of domain movement was observed within *hsdS* genes belonging to the same homology group. For example, identical TRDs were observed in all three homology group A *hsdS* genes at both TRD positions (i.e., TRD1 or TRD2) in different strains (e.g., TRD M), indicating that recombination between has taken place (fig. 3*c*). However, no TRDs are shared by *hsdS* homology group A and B, and regions adjacent to their TRDs have limited nucleotide identity (30–49%), suggesting domain movement between the two homology groups is unlikely.

Phylogenetic analysis of all Type I R-M system TRD sequences showed that two TRDs were found only in RB1 lineage strains, seventeen TRDs were found only in RB2/3 lineage strains, and two TRDs were found only in strains of divergent lineage. However, five TRDs (TRD A, B, D, S, and U) were found in strains from multiple phylogenetic lineages alongside lineage specific TRDs, suggesting recombination between lineages has occurred at the TRD level. In addition, TRDs A and B were also found co-located within the same *hsdS* gene in RB1 (e.g., 25240) and RB2/3 (e.g., A9) lineage strains, suggesting recombination occurs between *M. catarrhalis* phylogenetic lineages at the whole *hsdS* gene as well (supplementary fig. S2, Supplementary Material online).

### Variability within Type III R-M Systems

**Fig. 4.**
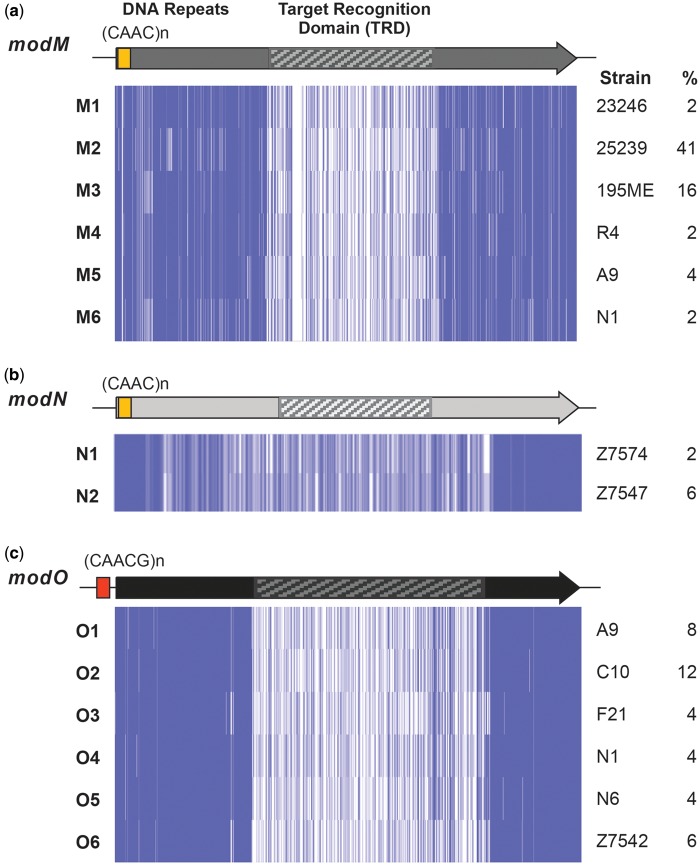
—Schematic of genes encoding phase-variable Type III DNA methyltransferases. (*a*) *modM*, (*b*) *modN*, and (*c*) *modO*. Type III DNA methyltransferase alleles (*modM1-6*, *modN1-2*, *modO1-6*) were aligned in ClustalW and visualized with JalView. Identical nucleotides are shown as vertical lines, with identity over a run of nucleotides shown as dark purple (>80% identity), light purple (>50%), or white (<50% identity or a gap). *Moraxella catarrhalis* representative strains that define each methyltransferase allele are indicated to the right of the alignment. Alleles were classified on the basis of ≥95% nucleotide identity of the target recognition domain (TRD) to one of these sequences. The proportion of strains (%) containing each methyltransferase allele in the 51 publicly available *M. catarrhalis* genomes is indicated on the far right. The locations of the 5′-(CAAC)_*n*_-3 repeats in the open reading frame (ORF) of *modM* and *modN*, and the 5′-(CAACG)_*n*_-3 repeat region upstream of the *modO* ORF, are indicated.

Type III R-M systems consist of a restriction endonuclease (Res) and a DNA methyltransferase (Mod) that contains the TRD and determines the recognition sequence of the system. Four putative Type III R-M systems were identified in this study: Mca25239IP at locus 1, McaC031IP at locus 10, Mca25239IIP at locus 11, and the previously discussed Mca25239ORF1708P at both locus 5 and 12 (fig. 1). We previously reported that the Type III DNA methyltransferase of Mca25239IP (*modM*) undergoes phase variation, mediated by a 5′-(CAAC)_*n*_-3′ tetranucleotide repeat tract in its ORF, and is an epigenetic regulator of a phasevarion (Seib et al. 2002; Blakeway et al. 2014). Three different *modM* alleles were identified: *modM1* (*mca23246IPmod*) (Seib et al. 2002), *modM2* (*mca25239Imod*) (Blakeway et al. 2014), and *modM3* (*mca195Imod*) (Tan et al. 2017) that differ in their variable TRD and methylate different target sequences (Blakeway et al. 2014). In this study we have identified three additional *modM* alleles, *modM4* (*mcaR4IPmod*), *modM5* (*McaS11IIPmod*), and *modM6* (*mcaN1IPmod*). Nucleotide sequence alignments of *modM1-6* showed that the conserved N and C-terminal regions share 91–97% nucleotide identity, whereas the TRD shares only 36–50% identity between alleles (fig. 4*a*). In addition to the overall association of R-M system type (Type I or Type III) at locus 1 with phylogenetic lineage (as described above), a significant association between specific *modM* alleles and lineage was observed. The *modM2* allele is exclusively associated with the RB1 lineage (present in 21/24 RB1 vs 0/9 RB2/3 strains; *P* ≤ 0.0001) while *modM3* is more commonly associated with the RB2/3 lineage (present in 3/24 RB1 vs 5/9 RB2/3 strains; *P* = 0.0201). The rare alleles *modM4*, *modM5*, and *modM6* were found exclusively in RB2/3 strains, whereas *modM1* was found in only one strain, Z7574, belonging to the divergent group.

One other phase-variable Type III DNA methyltransferase, *mca23246IIPmod* (*modN*) that also contains a 5′-(CAAC)_*n*_-3′ tetranucleotide repeat tract in its ORF was previously identified in *M. catarrhalis* 23246 (*mca23249IIPmod*, *modN1*) and was thought to exist solely in this strain (Seib et al. 2002). However, *modN* was found at locus 11 in four additional strains of the divergent lineage in this study (fig. 1), and a second *modN* allele (*mcaZ7547IIPmod*, *modN2*) was identified that differs in its central TRD (fig. 4*b*). In addition, we have identified a novel potentially phase variable Type III DNA methyltransferase at locus 10, *mcaC031IPmod* (which we propose be called *modO*) (fig. 2). The *modO* gene was found in 19 strains and has a 5′-(CAACG)_*n*_-3′ pentanucleotide repeat tract upstream of its annotated ORF. Six alleles of *modO* were found, which have 99% nucleotide identity in the N and C-terminal regions and 35–54% nucleotide identity in the TRD: *modO1* (*mcaA9IPmod*), *modO2* (*mcaC10IPmod*), *modO3* (*mcaF21IIPmod*), *modO4* (*mcaN1IPmod*), *modO5* (*mcaN6IPmod*), and *modO6* (*mcaZ7542IPmod*) (fig. 4*c*). Nucleotide sequence alignments of all *mod* genes showed that TRDs were not shared between loci and no homologous sequences were present, suggesting that unlike the Type I R-M systems, recombination and domain movement does not occur between Type III R-M system loci in *M. catarrhalis*.

### TRD Homologs in Other Bacterial Species

**Fig. 5.**
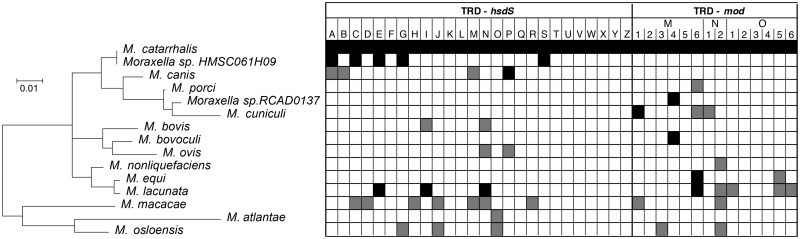
—Distribution of Type I and Type III R-M system TRD homologs in the *Moraxella* genus. Presence/absence matrix of Type I (*hsdS*) and Type III (*mod*) R-M system TRDs in fifteen *Moraxella* species. Each row represents one *Moraxella* species and each column represents one TRD. Black squares: Homologs with >90% amino acid identity. Grey Squares: Homologs with 44–85% amino acid identity. White squares: Absence of the TRD system in the species.

In order to investigate the origin of *M. catarrhalis* TRD sequences, we searched for *hsdS* and *mod* TRD homologs in other *Moraxella* species and distantly related genera. Homologues of *hsdS* TRDs with a high *e-*value (1*e*–100 or greater) in conjunction with high amino acid sequence identity (>90%) were found for TRDs A, C, E, G, and S in the closely related *Moraxella* sp. *HMSC061H09*, and for TRDs E, I and N in *Moraxella lacunata*, a commensal *Moraxella* species that occupies the same niche (fig. 5, supplementary table S2, Supplementary Material online). Homologues of *mod* TRDs were found in other *Moraxella* species for *modM1* (*Moraxella cuniculi*), *modM4* (*Moraxella* sp. *RCAD0137* and *Moraxella bovoculi*), and *modM6* (*Moraxella equi* and *Moraxella lacunata*) (fig. 5, supplementary table S3, Supplementary Material online). Homologs in other genera with high amino acid identity were found for *hsdS* TRD G in *Cyanobacterium* sp. *IPPASB-1200* (89%), *hsdS* TRD H in *Weeksella* sp. *HMSC059D05* (96%), and *modM3* in *Bibersteinia trehalosi* (99%) (supplementary tables S2 and S3, Supplementary Material online). Homologs with high *e-*values and moderate sequence identity (44–85%) were also observed, and these potentially constitute novel TRDs in other species (fig. 5, supplementary tables S2 and S3, Supplementary Material online).

### Distribution of locus 1 R-M systems in geographically diverse panels of *M. catarrhalis* Isolates

**Fig. 6.**
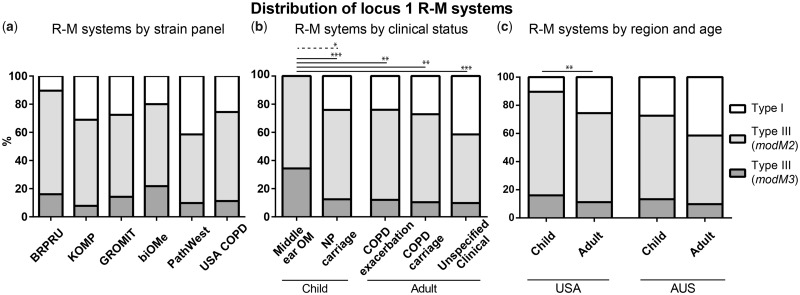
—Distribution of locus 1 mutually exclusive Type I and Type III R-M systems in diverse panels of *Moraxella catarrhalis* isolates. The distribution of Type I and Type III R-M systems (*modM2* or *modM3*) in six panels of *M. catarrhalis* isolates is shown by (*a*) strain panel (see table 2 for panel details), (*b*) clinical status, and (*c*) region and age. NP, nasopharyngeal; OM, otitis media; COPD, chronic obstructive pulmonary disease. Solid line; comparison of proportion of isolates containing a Type I or Type III R-M system. Dashed line: Comparison of proportion of strains containing the Type III DNA methyltransferase *modM2* or *modM3* allele. *P ≤ 0.05, **P ≤ 0.01, ***P ≤ 0.001, Fisher’s exact test.

We previously investigated the distribution of the *modM2* and *modM3* alleles in a collection of 81 nasopharyngeal carriage and OM-associated middle ear isolates from children and found a statistically significant association of *modM3* with OM isolates (Blakeway et al. 2014). This study has been expanded to include an additional 44 child nasopharyngeal carriage isolates in the BRPRU panel, and 424 isolates from five geographically, temporally, and clinically diverse panels of *M. catarrhalis*, totaling 549 isolates (table 2, fig. 6*a*). We also investigated the distribution of the mutually exclusive Type I R-M system at locus 1 in addition to the *modM2* and *modM3* alleles. The Type I and Type III R-M systems were found in 24% (133/549) and 76% (416/549) of strains, respectively. A statistically significant difference in the presence of the Type I or Type III R-M system was observed between child nasopharyngeal carriage and OM-associated middle ear isolates. The Type I and Type III R-M systems were present in 24% (91/378) and 76% (287/378) of child nasopharyngeal carriage isolates, respectively. Interestingly, middle ear isolates from children with OM (32/32) always contain an allele of the Type III ModM R-M system, with the Type I system never found (*P* = 0.0005) (fig. 6*b*). The Type I R-M system was overrepresented in USA isolates from adults compared with children, 26% (25/98) versus 10% (13/125), respectively (*P* = 0.0038) (fig. 6*c*). A similar trend was also observed in Australian isolates; however, the difference was not statistically significant; 41% (17/41) versus 27% (78/285) in adults versus children, respectively (*P* = 0.0685).

In the 416 strains containing the Type III R-M system, *modM2* was the most prevalent allele, present in 82% of isolates overall (73–89% in individual panels), followed by *modM3* which was present in 18% of total isolates (11–27% in individual panels). The *modM1*, *modM4*, *modM5*, and *modM6* alleles were rare and not found in any strains in the six panels. Child nasopharyngeal carriage and OM-associated middle ear isolates were distributed between four panels of isolates; the biOMe panel (Perth, Australia) (Lappan et al. 2018), the GROMIT panel (Perth, Australia) (Wiertsema et al. 2011), the Kalgoorlie Otitis Media Research Project (KOMRP) panel (Kalgoorlie-Boulder, Australia) (Lehmann et al. 2008), and the previously reported BRPRU panel (Ohio, USA) (Blakeway et al. 2014). When all four panels of child isolates were combined, a statistically significant association of the *modM3* allele with OM-associated middle ear isolates (34%; 11/32) compared with nasopharyngeal carriage isolates (16%; 47/287) was observed (*P* = 0.0262) (fig. 6*b*). Consistent with previous results, this association was observed in the expanded BRPRU panel (middle ear: 47% [8/17] vs nasopharynx: 13% [12/95]; *P* = 0.0024). However, no statistically significant association was found between either *modM2* or *modM3* and nasopharyngeal carriage or OM-associated middle ear isolates in the biOMe panel (*P* = 0.6625) or the GROMIT panel (*P* = 0.3380). No significant difference was observed in the distribution of R-M systems or *modM* alleles in child nasopharyngeal isolates regardless of the patient’s prior/current OM status (i.e., nasopharyngeal isolates from children with a history of OM or concurrent OM at the time of isolation versus healthy control children without prior OM). Similarly, no difference was observed between strains isolated from COPD patient sputum samples during an exacerbation versus periods of stable colonization (*P* > 0.05).

## Discussion

R-M systems are ubiquitous in bacteria, where they have fundamentally been associated with defense of the cell from incoming DNA, such as from bacteriophages (Bickle and Kruger 1993). The presence or absence of particular R-M systems has been associated with phylogenetically distinct clades in bacteria such as *N. meningitidis* (Budroni et al. 2011), *Staphylococcus aureus* (Roberts et al. 2013), and *Burkholderia pseudomallei* (Nandi et al. 2015). Furthermore, phase variable Type I and Type III R-M systems have been associated with epigenetic regulation in systems called phasevarions (Atack et al. 2018). All currently characterized phasevarions contain genes important for human infection, and genes that encode potential vaccine candidates (Tan, Atack, et al. 2016; Atack et al. 2018). Epigenetic gene regulation through the methylation of specific DNA sequences by solitary (or orphan) methyltransferases that are not associated with R-M systems, such as Dam, have also been described in a number of bacterial species (reviewed in Casadesus and Low 2006; Marinus and Casadesus 2009). In this study we have demonstrated that the pangenome of *M. catarrhalis* encodes 18 putative R-M systems, and 1 orphan Type I R-M system *hsdS* subunit. Twelve loci were identified where R-M systems are variably present between strains and replace other genes on the chromosome. Our investigation also shows that distinct R-M systems are associated with specific lineages of *M. catarrhalis*. In addition, we have provided a detailed analysis of allelic variation of Type I and Type III R-M systems and described the mobility of their variable TRDs. We have also identified a new phase variable Type III DNA methyltransferase (ModO), which may further increase the complexity of the *M. catarrhalis* methylome and impact *M. catarrhalis* pathobiology and vaccine development.

The *M. catarrhalis* species is comprised of two closely related phylogenetic lineages, RB1 and RB2/3 (Wirth et al. 2007; Earl et al. 2016). RB1 lineage strains have been associated with disease, and increased adherence to airway epithelial cells and serum resistance has been previously reported among RB1 strains compared with RB2/3 lineage strains (Bootsma et al. 2000; Wirth et al. 2007; Earl et al. 2016). Although comparative analysis of the core genomes of each lineage found 33 and 49 gene clusters unique to RB1 and RB2/3 lineage strains, respectively, which mainly encode hypothetical proteins of unknown function or phosphate metabolism proteins, no lineage specific virulence factors have been identified that account for the difference in virulence traits. Our data show a clear distribution of R-M systems in *M. catarrhalis* along phylogenetic lines (e.g., the putative Type IV R-M system [Mca25239MrrP at locus 4] is present ubiquitously in RB1 strains but absent from RB2/3 strains). Consistent with our results, a prior study by Earl et al. (2016) found a gene cluster containing Mca25239MrrP and the hypothetical protein encoding gene MCR_1123 (BBH18 locus tag) was aligned with lineage, however the functions of these genes were unknown at the time. Mca25239MrrP may be analogous to the lineage associated *drg* gene in *N. meningitidis*, which encodes a modification-dependent restriction endonuclease and replaces the *dam* methylase in the ST-1, ST-4, ST-5, ST-32, and ST-41/44 lineages (Jolley et al. 2004), however this has yet to be confirmed. In addition, we also observed considerable deviation in the presence/absence of R-M systems in divergent strains compared with either the RB1 or RB2/3 lineage, further adding to accumulating evidence that the divergent strains are taxonomically distinct from *M. catarrhalis*.

It is unknown whether differences in R-M system possession are responsible for the population structure of *M. catarrhalis*, or are a consequence of the independent evolution of the phylogenetic lineages. R-M systems have been associated with the maintenance of speciation and intraspecies population structure by limiting horizontal gene transfer and homologous recombination between genomes with noncognate R-M systems, while permitting the genetic flux between bacteria containing cognate R-M systems (Oliveira et al. 2016). Experimental data elucidating the roles of *M. catarrhalis* R-M systems in horizontal gene transfer is lacking, but horizontal gene transfer has been shown to be more extensive within the RB1 and RB2/3 lineages than between them (Earl et al. 2016). In addition, it has been observed that plasmid DNA is acquired more readily by an untransformed clone of the same strain that the plasmid was propagated in, rather than a heterogeneous strain, and it was hypothesized that this is due to differential R-M systems (Wang and Hansen 2006). Our data demonstrate that there are a sizeable number of R-M systems present in *M. catarrhalis* that may contribute to clade specific evolution. As only 51 genome sequences of *M. catarrhalis* are currently publicly available, there is also potential for further novel R-M systems to be identified as more genomes become available. In addition, novel *M. catarrhalis* R-M systems and subunits might not have been identified if they share little similarity with known R-M systems and were unannotated in the genomes examined, and therefore this work may not represent the full repertoire of R-M systems in *M. catarrhalis.*

Two putative Type I R-M systems were identified at locus 1 and locus 8, as well as one orphan *hsdS* gene at locus 6. The locus 1 Type I R-M system contains two *hsdS* genes in the majority of strains analyzed. Varying arrangements of Type I R-M systems have been observed in other bacteria; for example, in *S. aureus* two different *hsdM* and *hsdS* genes are located distally to the *hsdR*, and in *Lactococcus lactis* a second *hsdS* gene is located on a plasmid (reviewed in Furuta and Kobayashi 2013). It is unknown which of the three *hsdS* genes are expressed and utilized by the locus 1 Type I R-M system or if competition occurs. Two *hsdS* homology groups were identified based on the presence of homologous repeat regions adjacent to the TRDs that are unique to each homology group. Both Locus 1 *hsdS* genes and the distally located locus 6 orphan *hsdS* genes are part of the same homology group, and it is possible they may be part of the same system. Identical TRDs are found in both locus 1 *hsdS* genes and in the orphan *hsdS* at locus 6 in different strains, suggesting that domain movement occurs between the three homology group A *hsdS* genes. Domain movement of TRDs between Type I R-M system specificity subunits mediated by homologous recombination has been described in detail for other bacterial species (Furuta et al. 2011) and has been shown to alter the methylation specificity of the system (Furuta et al. 2014). In the Type I R-M system SpnD39III in *S. pneumoniae*, recombination of TRDs between the expressed *hsdS* gene and additional truncated *hsdS* genes within a strain alters the methylation specificity of the system and epigenetically alters gene expression in a manner corresponding to phase variation (Manso et al. 2014). We hypothesize that the TRDs in *M. catarrhalis hsdS* genes can also recombine within a single strain over successive generations and this warrants investigation, as this could further increase the complexity of the *M. catarrhalis* methylome and the number of genes under control of epigenetic regulation.

Phase-variable Type III DNA methyltransferases in *M. catarrhalis* (Blakeway et al. 2014), *N. meningitidis* (Jen et al. 2014), *N. gonorrhoeae* (Srikhanta et al. 2009), and *H. influenzae* (Atack et al. 2015) have been shown to act as epigenetic regulators of gene expression and differentially affect the virulence of these bacteria. We previously identified three *modM* alleles (*modM1-3*) and it was shown that ModM2 and ModM3 methylate different target sequences, 5′-GAR^m6^AC-3′ and 5′-AC^m6^ATC-3′, respectively (Blakeway et al. 2014). We have identified three additional *modM* alleles (*modM4-6*) in this study that contain different TRDs than the previously characterized alleles and are also predicated to methylate different target sequences. A second phase variable Type III DNA methyltransferase, *modN*, was previously identified in an atypical strain more closely related to *Moraxella canis* than *M. catarrhalis* (Seib et al. 2002; Juni and Bovre 2007). In this study, we have identified an additional *modN* allele (*modN2*) which was also found in atypical strains of *M. catarrhalis*. In addition, a novel Type III DNA methyltransferase (ModO), with six alleles (*modO1-6)* was identified in this study and is part of the locus 10 R-M system McaC031IP. Unlike *modM* and *modN*, that contain simple sequence repeats in their ORF that mediate phase variation at the translational level, *modO* contains a 5′-CAACG_(__*n*__)_-3′ repeat upstream of its ORF, which hypothesize will mediate phase variation at the transcriptional level. We predict that ModM1-6, ModN1-2, and ModO1-6 will each methylate different target sequences and regulate a different set of genes, that is, control different phasevarions, but this requires experimental confirmation. The presence of up to three independently switching phasevarions in individual *M. catarrhalis* strains is reminiscent of some *Neisseria* strains, that also contain up to three independently phase-variable methyltransferases (ModA, ModB, ModD, Seib et al. 2015), and would further complicate vaccine development due exponentially increasing the number of phase variable genes.

Our study of locus 1 in six distinct panels of *M. catarrhalis* isolates (549 isolates in total) demonstrated that the Type III R-M system (*modM*) was the most common across the six panels, occurring in 76% of isolates. Interestingly, all OM-associated middle ear isolates contained a Type III R-M system *modM* gene at locus 1 with the Type I system never occurring in these isolates. This suggests that strains containing a nonphase-variable Type I R-M system may be attenuated in their ability to infect or persist in the middle ear when compared with strains containing a phase variable Type III *modM* gene. This may be reflective of phylogenetic lineage as genome strains containing the Type I R-M system are associated with the less virulent RB2/3 lineage. Analysis of the distribution of *modM* alleles in these 549 isolates revealed that *modM2* was the most common allele, present in 82% of isolates, while *modM3* was found in the remaining 18% of isolates. When isolates were split by clinical manifestation, *modM3* was found in child OM-associated middle ear isolates at a significantly higher frequency than child nasopharyngeal carriage isolates. Although consistent with our previous study, wherein *modM3* was associated with child OM-associated middle ear isolates versus nasopharyngeal carriage isolates in the BRPRU panel (Ohio, USA) (Blakeway et al. 2014), this association was not observed in the biOMe (Perth, Australia) (Lappan et al. 2018) or GROMIT (Perth, Australia) (Wiertsema et al. 2011) panels. This discrepancy may be due to potential geographical differences in circulating strains or differences in presentation or severity of OM among study populations.

The data presented in this study show that a diverse range of systems exist in *M. catarrhalis*, and that many of these show associations with the phylogeny of the species. From these data, it is tempting to speculate that the acquisition of lineage associated R-M systems has shaped the *M. catarrhalis* population structure, potentially contributing to the differing genomic content, and by extension, the pathogenic potential of the *M. catarrhalis* phylogenetic lineages. In addition, a number of highly variable R-M systems that exhibit allelic variation, and are potentially phase variable were identified in *M. catarrhalis*. These novel R-M systems may contribute to pathogenesis if they facilitate epigenetic regulation in a manner analogous to the previously described ModM2 phasevarion (Blakeway et al. 2014), but additional experimental analysis is needed to support this. However, our data do suggest ModM strains are more frequently associated with OM than the Type I R-M containing strains in our surveyed collections.

## Supplementary Material

Supplementary Data
