# Supplementary material for: Moraxella catarrhalis Restriction–Modification Systems Are Associated with Phylogenetic Lineage and Disease
Source: Genome Biol Evol. 2018 Oct 18;10(11):2932–46. doi: 10.1093/gbe/evy226 (PMC6241649; doi:10.1093/gbe/evy226)
Supplement: Supplementary Data [file evy226_supp.pdf]

Moraxella catarrhalis Restriction-Modification Systems are Associated with Phylogenetic Lineage and Disease

Luke V. Blakeway[1], Aimee Tan[1], Rachael Lappan[2,3], Amir Ariff[2], Janessa L. Pickering[3,4], Christopher S. Peacock[2,3], Christopher C. Blyth[3,5,6,7], Charlene M. Kahler[2,3], Barbara J. Chang[2], Deborah Lehmann[3,4], Lea-Ann S. Kirkham[3,4], Timothy F. Murphy[8], Michael P. Jennings[1], Lauren O. Bakaletz[9], John M. Atack[1], Ian R. A. Peak[1,10] and Kate L. Seib[1,*]

Supplementary Tables S1-3 and Figures S1-2

Table S1: Primers used in this study

Primer	Sequence	Description
Multi-F	5'-GATGGCGTGATATTTATCAGTATTGATG-3'	Conserved *modM* forward primer upstream of variable target recognition domain (TRD)
Multi M1	5'-AGTACAAAGCTTCTTGATAATACAGCTC-3'	Reverse primer specific for *modM1* allele TRD
Multi M2	5'-CAGCCGAATAACCTTGAGTAGATG-3'	Reverse primer specific for *modM2* allele TRD
Multi M3	5'-CAAGGTTTTGGCTACTTTTCCTCG-3'	Reverse primer specific for *modM3* allele TRD
T1 mod F	5'-GGCAAATCGCCAACGATGTCAG-3'	Type I R-M system methyltransferase forward primer
T1 mod R	5'-CCTGAACCACAAGCAGGGTC-3'	Type I R-M system methyltransferase reverse primer
T1 res F	5'-GCCTGATAAAGCAGGCGTGATAG-3'	Type I R-M system restriction endonuclease forward primer
T1 res R	5'-CGTTTTCGCTGGCGTATCCTC-3'	Type I R-M system restriction endonuclease reverse primer

Table S2. BLASTx hits of Type I specificity subunit TRDs (top ten hits for each TRD

TRD	Description	Max score	Query cover	E value	Identity	Accession
A	*Moraxella sp.HMSC061H09*	245	99%	6.0E-79	100%	WP_070472555.1
	Haemophilus pittmaniae	197	99%	1.0E-62	79%	WP_007243555.1
	Aggregatibactersp.oraltaxon458	200	99%	3.0E-61	79%	WP_021615413.1
	Haemophilus pittmaniae	199	99%	9.0E-61	79%	WP_095176994.1
	Gallibacterium genomosp.3	198	99%	1.0E-60	80%	WP_065238990.1
	Aggregatibacter actinomycetemcomitans	198	99%	2.0E-60	81%	WP_005578267.1
	MULTISPECIES:Haemophilus	197	99%	3.0E-60	79%	WP_005639462.1
	Haemophilus quentini	197	99%	4.0E-60	79%	WP_070047000.1
	Haemophilus parainfluenzae	197	99%	4.0E-60	79%	WP_049376382.1
	Escherichia coli	194	97%	5.0E-60	81%	WP_106408229.1
B	*Moraxella canis*	167	97%	1.0E-48	67%	WP_078255377.1
	Moraxella canis	167	97%	2.0E-48	67%	WP_114799836.1
	Neisseria mucosa	161	97%	3.0E-48	63%	WP_107986692.1
	Neisseria cinerea	161	97%	3.0E-48	63%	WP_111726625.1
	Neisseria sicca	159	97%	3.0E-47	61%	WP_105165189.1
	Neisseria mucosa	159	97%	4.0E-47	61%	WP_107145094.1
	Alistipes sp.AL-1	154	97%	3.0E-45	68%	WP_032135130.1
	Pasteurella multocida	157	97%	2.0E-44	63%	WP_080940671.1
	Pasteurella multocida	157	97%	2.0E-44	63%	WP_015690924.1
	Lachno clostridium sp.An131	150	97%	7.0E-44	60%	WP_087165661.1
C	*Moraxella sp.HMSC061H09*	245	99%	2.0E-78	100%	WP_070472548.1
	Klebsiella pneumoniae	189	98%	7.0E-60	77%	WP_080878510.1
	Escherichia coli	191	98%	7.0E-60	78%	WP_112038435.1
	Leptospira kmetyi	196	99%	2.0E-59	77%	WP_010575851.1
	Leptospira kirschneri	194	99%	5.0E-59	77%	WP_020762562.1
	Klebsiella pneumoniae	189	98%	9.0E-59	77%	WP_077263917.1
	MULTISPECIES:Marinobacter	192	98%	6.0E-58	79%	WP_023011491.1
	Pseudomonas sp.EGD-AKN5	191	99%	1.0E-57	77%	WP_058487953.1
	Escherichia coli	191	98%	1.0E-57	78%	WP_096296258.1
	Escherichia coli	191	98%	2.0E-57	78%	WP_107127165.1
D	*Moraxella macacae*	182	99%	1.0E-53	73%	WP_009767330.1
	Neisseria elongata	176	99%	1.0E-53	70%	WP_082158248.1
	Thorsellia anophelis	181	99%	2.0E-53	73%	WP_093322588.1
	Wohlfahrtiimonas chitiniclastica	179	99%	1.0E-52	72%	WP_094493105.1
	Acidithiobacillus thiooxidans	178	99%	2.0E-52	69%	WP_065974753.1
	Acidithiobacillus albertensis	177	99%	7.0E-52	69%	WP_075321984.1
	Acidithiobacillus thiooxidans	177	99%	8.0E-52	69%	WP_081577508.1
	Morganella morganii	176	99%	2.0E-51	72%	WP_096747742.1
	Leptospira sp.E30	176	99%	4.0E-51	69%	WP_109022038.1
	Aeromonas salmonicida	174	99%	2.0E-50	70%	WP_059112975.1
E	*Moraxella lacunata*	278	99%	3.0E-91	99%	WP_062498863.1
	Moraxella sp.HMSC061H09	277	99%	1.0E-90	99%	WP_070472548.1
	Photorhabdus sp.HUG-39	225	99%	3.0E-70	81%	WP_113043819.1
	Escherichia coli	223	99%	5.0E-70	80%	WP_096838497.1
	Morganella morganii	224	99%	6.0E-70	81%	WP_079549445.1
	Runella zeae	218	99%	6.0E-70	78%	WP_084650916.1
	Morganella morganii	224	99%	8.0E-70	81%	WP_087770040.1
	Escherichia coli	223	99%	2.0E-69	80%	WP_089582146.1
	Escherichia coli	223	99%	2.0E-69	80%	WP_057080931.1
	MULTISPECIES:Xenorhabdus	223	99%	2.0E-69	80%	WP_047769912.1
F	*Thiobacillus thioparus*	201	99%	2.0E-61	79%	WP_018507927.1
	Dickeya zeae	199	98%	1.0E-60	79%	WP_038915178.1
	Stenotrophomonasmaltophilia	197	99%	4.0E-60	78%	WP_111150752.1
	Dickeyadadantii	197	98%	9.0E-60	78%	WP_038924819.1
	MULTISPECIES:Lebetimonas	195	96%	2.0E-59	79%	WP_035907639.1
	Chlorobaculum tepidum	191	99%	8.0E-57	76%	WP_010933538.1
	Gulbenkiania mobilis	187	99%	2.0E-56	75%	WP_054286757.1
	Thauera propionica	187	99%	3.0E-56	75%	WP_094268813.1
	Pseudomonas aeruginosa	187	99%	3.0E-56	75%	WP_073651904.1
	Porphyromonas gingivalis	179	97%	5.0E-55	74%	WP_081393753.1
G	*Moraxella sp.HMSC061H09*	222	99%	6.0E-70	100%	WP_070472555.1
	Cyanobacterium sp.IPPASB-1200	202	99%	5.0E-62	89%	WP_083260459.1
	Acinetobacter sp.ACNIH1	199	99%	1.0E-60	84%	WP_104426540.1
	Photorhabdus australis	198	99%	1.0E-60	84%	WP_036770802.1
	Photorhabdus australis	198	99%	1.0E-60	84%	WP_065822764.1
	Photorhabdus laumondii	197	99%	2.0E-60	83%	WP_114538532.1
	Photorhabdus laumondii	197	99%	2.0E-60	83%	WP_011148413.1
	Photorhabdus laumondii	197	99%	2.0E-60	83%	WP_109791867.1
	Xenorhabdus sp.NBAIIXenSa04	197	99%	3.0E-60	83%	WP_047685937.1
	Actinobacillus lignieresii	196	99%	5.0E-60	85%	WP_115590485.1
H	*Weeksella sp.HMSC059D05*	229	99%	3.0E-75	96%	WP_083299095.1
	Mannheimia haemolytica	194	99%	2.0E-61	79%	WP_115262446.1
	Mannheimia haemolytica	194	99%	3.0E-61	79%	WP_006252504.1
	Bacteroides coprosuis	197	99%	8.0E-60	80%	WP_006744891.1
	Mannheimia haemolytica	193	99%	2.0E-58	79%	WP_062627966.1
	Mannheimia haemolytica	193	99%	2.0E-58	79%	WP_020831153.1
	Mannheimia haemolytica	193	99%	3.0E-58	79%	WP_061887293.1
	Polaribacter gangjinensis	187	99%	7.0E-56	78%	WP_105045923.1
	Gallibacterium genomosp.3	183	99%	6.0E-55	73%	WP_065238990.1
	Haemophilus influenzae	172	99%	2.0E-52	67%	WP_082211360.1
I	*Moraxella lacunata*	275	99%	8.0E-90	98%	WP_062498863.1
	Moraxella lacunata	273	99%	2.0E-89	99%	WP_115246849.1

TRD	Description	Max score	Query cover	E value	Identity	Accession
N	*Moraxella lacunata*	244	99%	3.0E-78	100%	WP_115246849.1
	Neisseria meningitidis	201	99%	7.0E-64	79%	WP_050893130.1
	Neisseria meningitidis	201	99%	7.0E-64	79%	WP_115437084.1
	Neisseria meningitidis	201	99%	7.0E-64	79%	WP_082301664.1
	Neisseria meningitidis	201	99%	7.0E-64	79%	WP_101094598.1
	Neisseria meningitidis	201	99%	8.0E-64	79%	WP_002250111.1
	Neisseria meningitidis	201	99%	8.0E-64	79%	WP_002235659.1
	MULTISPECIES: Neisseria	201	99%	9.0E-64	79%	WP_002213928.1
	Neisseria meningitidis	201	99%	9.0E-64	79%	WP_050162546.1
	Neisseria meningitidis	201	99%	1.0E-63	79%	WP_002252708.1
O	*Haemophilus haemoglobinophilus*	222	99%	1.0E-68	69%	WP_115072418.1
	Enterococcus faecalis	200	96%	2.0E-60	67%	WP_113795148.1
	Enterococcus faecium	200	96%	3.0E-60	67%	WP_094899404.1
	Acetoanaerobium noterae	199	99%	5.0E-60	66%	WP_079588690.1
	Neisseria elongata	192	99%	3.0E-59	62%	WP_049251892.1
	Flavobacterium aurantiibacter	196	99%	6.0E-59	63%	WP_094487315.1
	Chitinophaga skermanii	195	99%	5.0E-58	64%	WP_111599288.1
	Paenibacillus durus	194	99%	6.0E-58	62%	WP_042209053.1
	Lysinibacillus massiliensis	194	99%	6.0E-58	62%	WP_036177514.1
	Trichococcus pasteurii	193	99%	2.0E-57	62%	WP_086942898.1
P	*Moraxella canis*	231	99%	1.0E-75	91%	WP_114799839.1
	Moraxella canis	232	99%	2.0E-73	91%	WP_078255375.1
	Moraxella canis	229	99%	2.0E-72	89%	WP_078255377.1
	Actinobacillus capsulatus	225	99%	6.0E-71	85%	WP_018652509.1
	Haemophilus paracuniculus	222	99%	3.0E-70	87%	WP_078235728.1
	Actinobacillus pleuropneumoniae	222	99%	7.0E-70	84%	WP_005596201.1
	Methylobacter tundripaludum	222	99%	1.0E-69	84%	WP_104422266.1
	Moraxella ovis	221	99%	3.0E-69	85%	WP_063514821.1
	Enterococcus mundtii	216	99%	2.0E-67	84%	WP_108144719.1
	Gallibacterium genomosp.3	216	99%	2.0E-67	83%	WP_065235157.1
Q	*Serratia ficaria*	194	99%	6.00E-61	75%	WP_083950327.1
	Cardiobacterium valvarum	184	99%	4.00E-57	71%	WP_083826237.1
	Escherichia coli	188	99%	6.00E-57	70%	WP_072315675.1
	Escherichia coli	189	99%	7.00E-57	72%	WP_108451335.1
	Cronobacter sakazakii	189	99%	2.00E-56	70%	WP_110792183.1
	Cronobacter sakazakii	189	99%	2.00E-56	70%	WP_105596552.1
	Escherichia coli	189	99%	3.00E-56	70%	WP_112923782.1
	Klebsiella pneumoniae	189	99%	3.00E-56	70%	WP_109262097.1
	Shewanella putrefaciens	188	99%	4.00E-56	69%	WP_014611659.1
	Escherichia coli	184	99%	4.00E-56	70%	WP_075700214.1
R	*Moraxella macacae*	243	99%	2.00E-77	81%	WP_009501175.1
	Mannheimia granulomatis	242	99%	2.00E-76	83%	WP_027074206.1
	Actinobacillus seminis	241	99%	4.00E-76	83%	WP_094946302.1
	Histophilus somni	240	99%	6.00E-76	83%	WP_075293868.1
	Histophilus somni	240	99%	9.00E-76	83%	WP_087449232.1
	Campylobacter concisus	200	98%	2.00E-60	66%	WP_107776421.1
	Brachyspira hyodysenteriae	197	96%	3.00E-59	67%	WP_047104848.1
	Streptococcus mitis	195	99%	2.00E-58	64%	WP_061424273.1
	Brachyspira pilosicoli	193	96%	8.00E-58	66%	WP_014936861.1
	Brachyspira pilosicoli	193	96%	1.00E-57	66%	WP_014936074.1
S	*Moraxella sp. HMSC061H09*	158	98%	1.00E-62	100%	WP_070473235.1
	Escherichia coli	132	98%	2.00E-50	84%	WP_105997262.1
	Chitinivibrio alkaliphilus	132	99%	5.00E-49	81%	WP_022637522.1
	MULTISPECIES: Enterobacteriaceae	128	98%	4.00E-48	80%	WP_001553855.1
	MULTISPECIES: Enterobacteriaceae	128	98%	5.00E-48	80%	WP_048266692.1
	Escherichia coli	128	98%	5.00E-48	80%	WP_112037710.1
	MULTISPECIES: Enterobacteriaceae	128	98%	5.00E-48	80%	WP_001617890.1
	Escherichia coli	128	98%	5.00E-48	80%	WP_042079866.1
	MULTISPECIES: Enterobacteriaceae	128	98%	5.00E-48	80%	WP_023142242.1
	Escherichia coli	128	98%	5.00E-48	80%	WP_089581154.1
T	*Proteus mirabilis*	197	197	2.00E-58	65%	WP_110706572.1
	Proteus mirabilis	196	96%	2.00E-58	65%	WP_004250449.1
	MULTISPECIES: Aeromonas	196	96%	3.00E-58	65%	WP_005300131.1
	Pantoea ananatis	195	96%	5.00E-58	66%	WP_052656065.1
	Aeromonas encheleia	194	96%	1.00E-57	65%	WP_042652836.1
	Aeromonas hydrophila	177	96%	6.00E-51	60%	WP_080688892.1
	Thiothrix caldifontis	177	96%	2.00E-50	60%	WP_093070280.1
	Burkholderia sp. AU4i	174	91%	4.00E-50	61%	WP_081712835.1
	Ralstonia solanacearum	173	95%	2.00E-49	58%	WP_081272534.1
	Arthrobacter sp. SRS-W-1-2016	167	96%	2.00E-47	58%	WP_078108152.1
U	*Campylobacter concisus*	193	97%	1.00E-58	73%	WP_087579744.1
	Campylobacter concisus	192	97%	4.00E-58	72%	WP_107713917.1
	Mycoplasma gallisepticum	184	96%	3.00E-57	68%	WP_011113945.1
	Campylobacter concisus	190	97%	5.00E-57	72%	WP_103609702.1
	Bacillus cereus	187	98%	1.00E-55	70%	WP_000792244.1
	Gallibacterium anatis	179	96%	3.00E-55	69%	WP_094933667.1
	Kandleria vitulina	178	98%	6.00E-55	66%	WP_080692698.1
	Lactococcus sp. DD01	184	98%	7.00E-55	68%	WP_082779235.1
	Gallibacterium anatis	177	96%	1.00E-54	68%	WP_018346289.1
	Gallibacterium anatis	178	96%	2.00E-54	68%	WP_039152775.1
V	*Neisseria elongata*	201	99%	9.00E-61	72%	WP_053090344.1
	Campylobacter concisus	178	98%	1.00E-52	64%	WP_103609702.1

	Leptospira interrogans	185	99%	5.0E-57	64%	WP_094188721.1
	Leptospira interrogans	185	99%	1.0E-54	64%	WP_000413321.1
	Leptospira interrogans	185	99%	1.0E-54	64%	WP_061231806.1
	Leptospira interrogans	184	99%	1.0E-54	65%	WP_017860629.1
	Leptospira interrogans	185	99%	1.0E-54	64%	WP_000413327.1
	Leptospira interrogans	184	99%	1.0E-54	65%	WP_000080670.1
	Leptospira interrogans	184	99%	2.0E-54	65%	WP_017861058.1
	Leptospira interrogans	184	99%	2.0E-54	64%	WP_000413328.1
**J**	*Mannheimia sp.10_1_50*	221	99%	6.0E-72	82%	WP_009293972.1
	Mannheimia haemolytica	229	99%	7.0E-72	84%	WP_020831153.1
	Mannheimia haemolytica	228	99%	7.0E-72	84%	WP_062627966.1
	Mannheimia haemolytica	228	99%	8.0E-72	84%	WP_061887293.1
	Mannheimia concisus	221	99%	9.0E-72	82%	WP_103578864.1
	Mannheimia haemolytica	220	95%	2.0E-71	85%	WP_050412810.1
	Paenibacillus odorifer	225	97%	2.0E-70	82%	WP_076224123.1
	Lascolabacillus massiliensis	223	98%	6.0E-70	79%	WP_053826540.1
	Bergeyella zoohelcum	216	96%	7.0E-70	80%	WP_002687895.1
	Vibrio anguillarum	223	97%	9.0E-70	81%	WP_088728543.1
**K**	*Neisseria subflava*	202	99%	5.0E-62	79%	WP_107723954.1
	Caedimonas varicaedens	189	99%	1.0E-59	73%	WP_062140202.1
	Riemerella anatipestifer	195	99%	3.0E-59	74%	WP_109474943.1
	Gilliamella apicola	190	99%	2.0E-58	73%	WP_081298739.1
	Haemophilus influenzae	193	99%	3.0E-58	74%	WP_112082530.1
	Snodgrassella alvi	192	99%	9.0E-58	74%	WP_100151927.1
	Gilliamella apicola	191	99%	1.0E-57	73%	WP_065589928.1
	Gilliamella apicola	191	99%	1.0E-57	74%	WP_081303181.1
	Actinobacillus ureae	191	99%	1.0E-57	74%	WP_115606724.1
	Haemophilus haemolyticus	191	99%	1.0E-57	74%	WP_046942422.1
**L**	*Spongiibactermarinus*	175	99%	1.0E-50	60%	WP_027873395.1
	Haemophilus influenzae	168	99%	3.0E-50	60%	WP_080351266.1
	Acidithiobacillus thiooxidans	174	99%	3.0E-50	61%	WP_024894395.1
	Acidithiobacillus thiooxidans	173	99%	6.0E-50	61%	WP_010640867.1
	Gilliamella apicola	173	99%	6.0E-50	61%	WP_065585600.1
	Gilliamella apicola	173	99%	6.0E-50	61%	WP_065636726.1
	Gilliamella apicola	171	99%	2.0E-49	60%	WP_065575997.1
	Aeromonas allosaccharophila	170	99%	7.0E-49	59%	WP_042060671.1
	Haemophilus influenzae	169	99%	2.0E-48	60%	WP_105872135.1
	Haemophilus influenzae	168	99%	2.0E-48	60%	WP_112111438.1
**M**	*Providenciasp.WCHPr000369*	182	97%	7.0E-56	84%	WP_102139452.1
	Enterococcus mundtii	184	99%	4.0E-55	83%	WP_108173626.1
	Neisseria lactamica	179	99%	6.0E-55	80%	WP_096107643.1
	Flavobacterium columnare	184	99%	7.0E-55	85%	WP_088466690.1
	Escherichia coli	176	97%	1.0E-54	81%	WP_039026547.1
	Escherichia coli	176	97%	1.0E-54	81%	WP_072185841.1
	Neisseria lactamica	177	99%	2.0E-54	80%	WP_114936056.1
	Escherichia coli	176	97%	2.0E-54	81%	WP_109536579.1
	Escherichia coli	177	97%	2.0E-54	81%	WP_054623585.1
	Escherichia coli	175	97%	3.0E-54	81%	WP_000328773.1

	Campylobacter curvus	176	97%	8.00E-52	63%	WP_018135884.1
	Campylobacter concisus	175	98%	2.00E-51	63%	WP_109146486.1
	Yersinia mollaretii	161	95%	2.00E-45	62%	WP_054878682.1
	Ignatzschineria sp. UAE-HKU60	159	98%	1.00E-44	58%	WP_109220058.1
	Escherichia coli	159	95%	2.00E-44	58%	WP_044697430.1
	Escherichia coli	158	95%	3.00E-44	58%	WP_000228012.1
	Enterobacter cloacae complex sp.	155	95%	3.00E-43	60%	WP_107535643.1
	Methanomethylovorans hollandica	144	98%	1.00E-38	53%	WP_015324748.1
**y**	*Undibacterium pigrum*	229	98%	3.00E-71	76%	WP_110253574.1
	Vibrio cholerae	226	96%	1.00E-69	75%	WP_071187857.1
	Escherichia coli	223	96%	3.00E-69	78%	WP_101917261.1
	Escherichia coli	223	96%	4.00E-69	78%	WP_052415654.1
	Leptospira meyeri	218	99%	1.00E-67	72%	WP_004789098.1
	Ignatzschineria indica	218	99%	5.00E-67	72%	WP_109235566.1
	Acinetobacter sp. Ver3	217	96%	2.00E-66	71%	WP_051586570.1
	Vibrio cholerae	216	96%	3.00E-66	71%	WP_095467530.1
	Klebsiella pneumoniae	215	96%	5.00E-66	73%	WP_114670167.1
	Shewanella baltica	215	96%	7.00E-66	70%	WP_107949522.1
**X**	*Glaciecola sp. KUL10*	176	97%	2.00E-50	57%	WP_110427271.1
	Edwardsiella piscicida	172	93%	5.00E-49	58%	WP_071819616.1
	Edwardsiella tarda	172	93%	5.00E-49	58%	WP_080545142.1
	Trabulsiella odontotermitis	171	98%	2.00E-48	56%	WP_049855668.1
	Yersinia kristensenii	166	99%	2.00E-46	55%	WP_050290238.1
	Enterobacter cloacae complex sp.	165	99%	2.00E-46	55%	WP_107535643.1
	Aeromonas veronii	163	93%	2.00E-45	58%	WP_088869043.1
	Sedimenticola thiotaurini	161	96%	2.00E-44	53%	WP_082117205.1
	Nitrosomonas nitrosa	144	99%	7.00E-39	47%	WP_107790358.1
	Marinobacter sp. N4	141	95%	2.00E-37	50%	WP_104320494.1
**Y**	*Neisseria mucosa*	218	98%	4.00E-67	75%	WP_107145908.1
	Campylobacter concisus	204	97%	3.00E-62	72%	WP_103637513.1
	Campylobacter concisus	204	97%	5.00E-62	72%	WP_103572274.1
	Campylobacter concisus	182	88%	2.00E-54	69%	WP_103622920.1
	Helicobacter pylori	177	99%	2.00E-53	62%	WP_100972737.1
	Helicobacter pylori	177	97%	3.00E-53	63%	WP_079370692.1
	Helicobacter pylori	177	99%	4.00E-53	62%	WP_108538651.1
	Helicobacter pylori	176	99%	6.00E-53	62%	WP_078279229.1
	Aeromonas sp. ARM81	182	97%	6.00E-53	64%	WP_114523358.1
	Helicobacter pylori	176	99%	7.00E-53	61%	WP_001953187.1
**Z**	*Snodgrassella alvi*	233	99%	2.00E-73	82%	WP_100151927.1
	Snodgrassella alvi	231	99%	1.00E-72	81%	WP_100150160.1
	Gallibacterium anatis	230	99%	2.00E-72	80%	WP_039091562.1
	MULTISPECIES: Paenibacillus	229	99%	6.00E-72	81%	WP_028542004.1
	Leptospira noguchii	225	99%	3.00E-70	78%	WP_004457293.1
	Leptospira noguchii	225	99%	3.00E-70	78%	WP_061245648.1
	Leptospira kirschneri	224	99%	4.00E-70	78%	WP_016561176.1
	Leptospira borgpetersenii	224	99%	5.00E-70	78%	WP_061209400.1
	Leptospira kirschneri	224	99%	6.00E-70	78%	WP_016751639.1
	MULTISPECIES: Leptospira	224	99%	6.00E-70	78%	WP_002760061.1

Table S3. BLASTx hits of Type III DNA methyltransferase TRDs (top ten hits for each TRD are listed)

TRD	Description	Max score	Query cover	E value	Identity	Accession
**M1**	*Moraxella cuniculi*	365	98%	7.0E-123	92%	WP_078310085.1
	Moraxella cuniculi	365	98%	2.0E-121	92%	WP_076555477.1
	Gallibacterium anatis	317	99%	4.0E-103	79%	WP_013746109.1
	Neisseria meningitidis	311	98%	5.0E-100	78%	WP_096100003.1
	Moraxella macacae	306	98%	2.0E-98	76%	WP_009767428.1
	Megamonas hypermegale	305	99%	5.0E-98	75%	WP_027889005.1
	Campylobacter coli	304	98%	8.0E-98	74%	WP_075441003.1
	Pelistega indica	302	96%	2.0E-97	77%	WP_081710597.1
	Clostridioides difficile	303	99%	3.0E-97	74%	WP_077745961.1
	Pelistega sp. MC2	302	96%	8.0E-97	77%	WP_083331897.1
**M2**	*Pasteurella multocida*	295	96%	4.0E-94	71%	WP_005756769.1
	Pasteurella multocida	295	96%	5.0E-94	71%	WP_016533629.1
	Rodentibacter rarus	285	96%	4.0E-87	70%	WP_077500275.1
	Haemophilus influenzae	284	96%	6.0E-87	69%	WP_114893168.1
	Rappaport israeli	262	99%	6.0E-83	65%	WP_072281377.1
	Glaesserella parasuis	265	99%	3.0E-82	64%	WP_081364047.1
	Campylobacter sp. P162	257	97%	5.0E-82	64%	WP_086318827.1
	Campylobacter lanienae	251	99%	5.0E-77	60%	WP_096014056.1
	Anaerosphaera sp. HMSC064C01	165	99%	7.0E-45	44%	WP_070599506.1
	Parvimonas sp. KA00067	161	99%	3.0E-44	42%	WP_082713881.1
**M3**	*Bibersteinia trehalosi*	362	99%	1.0E-120	88%	WP_015432567.1
	Desulfosporosinus sp. FKB	307	97%	8.0E-99	74%	WP_088226054.1
	Desulfosporosinus sp. FKA	306	97%	1.0E-98	74%	WP_088189609.1
	Desulfosporosinus acidiphilus	306	97%	1.0E-98	75%	WP_014826615.1
	Desulfosporosinus acididurans	305	97%	4.0E-98	75%	WP_047809141.1
	Haemophilus parainfluenzae	304	98%	9.0E-98	73%	WP_005696145.1
	Haemophilus parainfluenzae	305	98%	9.0E-98	73%	WP_080971680.1
	Haemophilus parainfluenzae	304	98%	1.0E-97	73%	WP_102704401.1
	Mobiluncus mulieris	293	99%	2.0E-93	70%	WP_103759143.1
	Leptotrichia sp. oral taxon 225	286	96%	7.0E-91	73%	WP_036071626.1
**M4**	*Moraxella bovoculi*	387	99%	3.0E-131	99%	WP_036365226.1
	Moraxella sp. RCAD0137	387	99%	8.0E-130	99%	WP_103033774.1
	Porphyromonadaceae bacterium COT	226	99%	5.0E-68	59%	WP_036826390.1
	Capnocytophaga sp. ChDC OS43	197	99%	1.0E-56	51%	WP_088594949.1
	Lactococcus lactis	195	99%	3.0E-56	51%	WP_101944589.1
	Dehalococcoides mccartyi	192	99%	6.0E-55	51%	WP_103908346.1
	Corynebacterium diphtheriae	186	99%	2.0E-52	45%	WP_003853194.1
	Corynebacterium minutissimum	177	99%	8.0E-51	46%	WP_115022228.1
	Corynebacterium minutissimum	176	99%	2.0E-50	46%	WP_052319707.1
	Corynebacterium sp. HMSC078H07	176	99%	2.0E-50	46%	WP_070669803.1
**M5**	*Clostridium perfringens*	251	99%	1.0E-76	58%	WP_110035132.1
	Clostridium perfringens	250	99%	2.0E-76	57%	WP_078209827.1
	Sneathia sanguinegens	250	99%	2.0E-76	55%	WP_066728972.1
	Streptobacillus ratti	238	99%	9.0E-72	54%	WP_072593630.1
	Eggerthia catenaformis	236	99%	1.0E-70	55%	WP_026624753.1
	Olsenella sp. An270	186	99%	2.0E-51	44%	WP_087225069.1
	Helicobacter canis	129	95%	3.0E-31	37%	WP_115011182.1
	Campylobacter upsaliensis	127	95%	1.0E-30	34%	WP_004276995.1
	Campylobacter upsaliensis	125	95%	5.0E-30	34%	WP_004274859.1
	Lactobacillus salivarius	124	97%	7.0E-29	35%	WP_113895797.1
**M6**	*Moraxella lacunata*	398	99%	2.0E-135	100%	WP_065256817.1
	Moraxella equi	380	99%	2.0E-128	95%	WP_079324856.1
	Moraxella equi	380	99%	1.0E-126	95%	WP_115236998.1
	Neisseria meningitidis	331	99%	8.0E-109	80%	WP_101127630.1
	Neisseria meningitidis	331	99%	1.0E-108	80%	WP_025459325.1
	Neisseria meningitidis	330	99%	2.0E-108	80%	WP_002241625.1
	Neisseria meningitidis	330	99%	2.0E-108	80%	WP_101127867.1
	Neisseria meningitidis	332	99%	6.0E-108	80%	WP_096112112.1
	Neisseria meningitidis	331	99%	7.0E-108	80%	WP_101120299.1
	Neisseria meningitidis	331	99%	1.0E-107	80%	WP_101123910.1
**N1**	*Haemophilus influenzae*	366	96%	1.0E-123	73%	WP_044330662.1
	Haemophilus influenzae	366	96%	1.0E-123	73%	WP_112070237.1
	Haemophilus influenzae	366	96%	1.0E-123	73%	WP_106404302.1
	Haemophilus influenzae	366	96%	1.0E-123	73%	WP_105877079.1
	Haemophilus influenzae	366	96%	1.0E-123	73%	WP_105873722.1
	Haemophilus influenzae	366	96%	1.0E-123	73%	WP_105887024.1
	Haemophilus influenzae	366	96%	1.0E-123	73%	WP_105894129.1
	Haemophilus influenzae	366	96%	2.0E-123	73%	WP_061716487.1
	Haemophilus influenzae	366	96%	2.0E-123	73%	WP_005686323.1
	Haemophilus influenzae	366	96%	2.0E-123	73%	WP_105873017.1
**N2**	*Streptococcus oralis*	551	99%	0.0E+00	71%	WP_084946013.1
	MULTISPECIES: Streptococcus	551	99%	0.0E+00	71%	WP_000806988.1
	Leptotrichia wadei	523	99%	9.0E-180	69%	WP_060918264.1
	Thermoflexibacter ruber	484	99%	2.0E-164	64%	WP_091549071.1
	Clostridiales bacterium KA00134	480	99%	8.0E-163	64%	WP_066537626.1
	Flavobacterium fontis	479	99%	1.0E-162	64%	WP_073365481.1
	Leptotrichia wadei	455	85%	8.0E-157	70%	WP_081617798.1
	Haemophilus influenzae	420	98%	6.0E-139	58%	WP_112081040.1
	Moraxella nonliquefaciens	420	98%	8.0E-139	58%	WP_067009844.1
	Haemophilus influenzae	392	99%	6.0E-131	55%	WP_114892802.1

TRD	Description	Max score	Query cover	E value	Identity	Accession
**O1**	*Moraxella lacunata*	442	100%	5.0E-149	75%	WP_115006712.1
	Lactobacillus iners	263	65%	6.0E-83	62%	WP_080550285.1
	Neisseria sp. HMSC064E01	248	98%	4.0E-75	47%	WP_083294158.1
	Aggregatibacter kilianii	249	100%	1.0E-74	45%	WP_109082808.1
	Geobacillus sp. WSUCF-018B	222	91%	3.0E-64	45%	WP_100664355.1
	Geobacillus thermodenitrificans	219	91%	3.0E-63	44%	WP_087959955.1
	Geobacillus sp. PA-3	219	91%	3.0E-63	44%	WP_060475780.1
	Parageobacillus thermoglucosidasius	216	91%	4.0E-62	44%	WP_073519227.1
	Parageobacillus thermoglucosidasius	215	91%	1.0E-61	44%	WP_042383172.1
	Geobacillus stearothermophilus	215	91%	2.0E-61	44%	WP_033015534.1
**O2**	*Capnocytophaga sp. oral taxon 326*	297	99%	1.0E-92	52%	WP_009750783.1
	Campylobacter concisus	291	99%	7.0E-91	52%	WP_103652489.1
	MULTISPECIES: Trichococcus	291	99%	1.0E-90	54%	WP_068560815.1
	Neisseria bergeri	275	100%	2.0E-84	50%	WP_107890413.1
	Enterococcus faecium	275	99%	2.0E-84	51%	WP_099097711.1
	Enterococcus faecium	274	99%	3.0E-84	51%	WP_073466471.1
	Neisseria bergeri	275	100%	3.0E-84	50%	WP_107818927.1
	Gramella flava	268	99%	9.0E-82	47%	WP_083645932.1
	Bacillus ginsengihumi	260	80%	5.0E-79	52%	WP_051476289.1
	Gracilibacillus timonensis	258	75%	5.0E-78	55%	WP_078060179.1
**O3**	*Clostridium novyi*	273	98%	2.0E-83	47%	WP_039249799.1
	Acinetobacter baumannii	254	87%	6.0E-76	52%	WP_109046184.1
	Bartonella queenslandensis	248	88%	1.0E-75	47%	WP_083868416.1
	Proteus sp. TJ1636	249	88%	8.0E-75	48%	WP_109396807.1
	Viridibacillus sp. OK051	250	88%	1.0E-74	48%	WP_100796992.1
	Acinetobacter baumannii	246	88%	3.0E-74	49%	WP_114161543.1
	Klebsiella pneumoniae	248	89%	1.0E-73	49%	WP_087799094.1
	Lactococcus fujiensis	226	88%	2.0E-65	43%	WP_096819171.1
	Lactococcus raffinolactis	226	88%	2.0E-65	43%	WP_003139707.1
	Viridibacillus arvi	225	91%	3.0E-65	43%	WP_053416983.1
**O4**	*Rodentibacter rarus*	394	96%	3.0E-130	65%	WP_077418050.1
	Neisseria sicca	308	98%	8.0E-98	49%	WP_107843267.1
	Neisseria weaveri	303	100%	8.0E-96	49%	WP_107855606.1
	Staphylococcus chromogenes	260	98%	2.0E-80	48%	WP_107364510.1
	Lactobacillus camelliae	246	99%	4.0E-73	45%	WP_056989867.1
	Lactobacillus sakei	245	99%	5.0E-73	44%	WP_056947062.1
	Dolosigranulum pigrum	235	95%	2.0E-71	46%	WP_111950628.1
	Campylobacter corcagiensis	160	98%	5.0E-41	36%	WP_034971544.1
	Thiomicrospira aerophila	150	100%	1.0E-37	34%	WP_006459402.1
	Rodentibacter heylii	146	99%	4.0E-36	36%	WP_077581730.1
**O5**	*Neisseria meningitidis*	488	100%	3.0E-170	79%	WP_082299901.1
	Neisseria meningitidis	488	100%	1.0E-169	79%	WP_079875826.1
	Neisseria meningitidis	488	100%	1.0E-169	79%	WP_072104357.1
	Neisseria meningitidis	489	100%	8.0E-169	79%	WP_080611275.1
	Neisseria meningitidis	489	100%	1.0E-168	79%	WP_014575393.1
	MULTISPECIES: Neisseria	489	100%	1.0E-168	79%	WP_047922166.1
	Neisseria meningitidis	489	100%	1.0E-168	79%	WP_101087827.1
	Neisseria gonorrhoeae	489	100%	2.0E-168	79%	WP_003689017.1
	Neisseria gonorrhoeae	488	100%	2.0E-168	79%	WP_050173535.1
	Neisseria meningitidis	488	100%	2.0E-168	79%	WP_101131695.1
**O6**	*Neisseria polysaccharea*	372	100%	5.0E-123	69%	WP_081456193.1
	Neisseria polysaccharea	372	100%	2.0E-122	69%	WP_115437506.1
	Neisseria meningitidis	369	100%	3.0E-121	69%	WP_101124415.1
	Neisseria lactamica	333	98%	1.0E-107	63%	WP_065425734.1
	Moraxella lacunata	326	100%	3.0E-105	61%	WP_062498748.1
	Clostridium sp. LS	223	99%	2.0E-65	46%	WP_008423295.1
	Caloramator quimbayensis	221	99%	2.0E-64	45%	WP_078696724.1
	Clostridium algidicarnis	192	99%	2.0E-53	44%	WP_104410678.1
	Clostridium kluyveri	191	99%	6.0E-53	41%	WP_073537112.1
	Rhodococcus sp. R1101	184	98%	9.0E-51	38%	WP_016932627.1

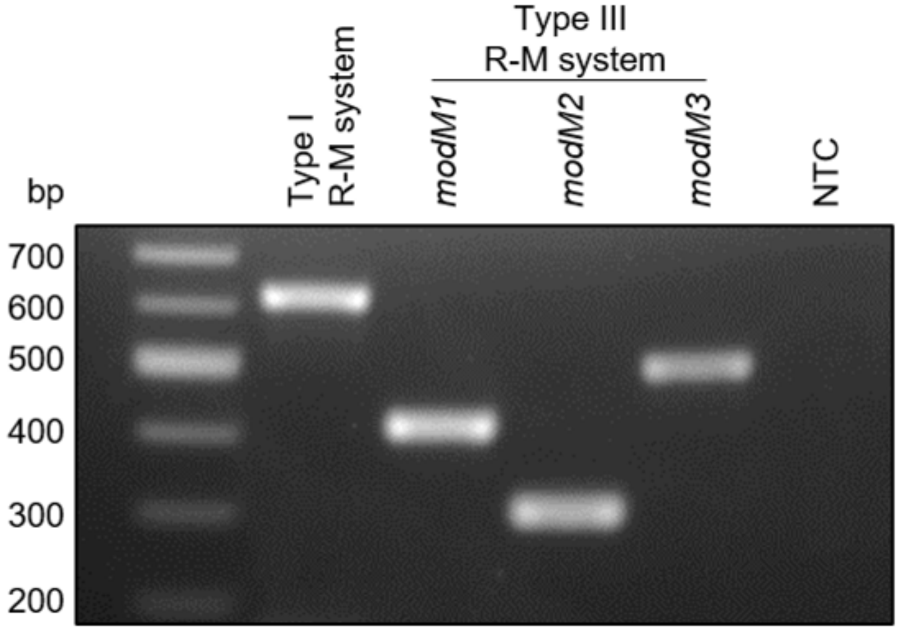

Figure S1: Restriction-modification (R-M) system screening by Multiplex PCR. A representative multiplex PCR reaction is shown. Different sized amplicons are generated depending on which of the mutually exclusive locus 1 restriction-modification (R-M) systems (Type I or Type III) or Type III DNA methyltransferase alleles are present in a strain. Type I R-M system: 600 bp, *modM1*: 400 bp, *modM2*: 300 bp, *modM3*: 500 bp. NTC = no template control.

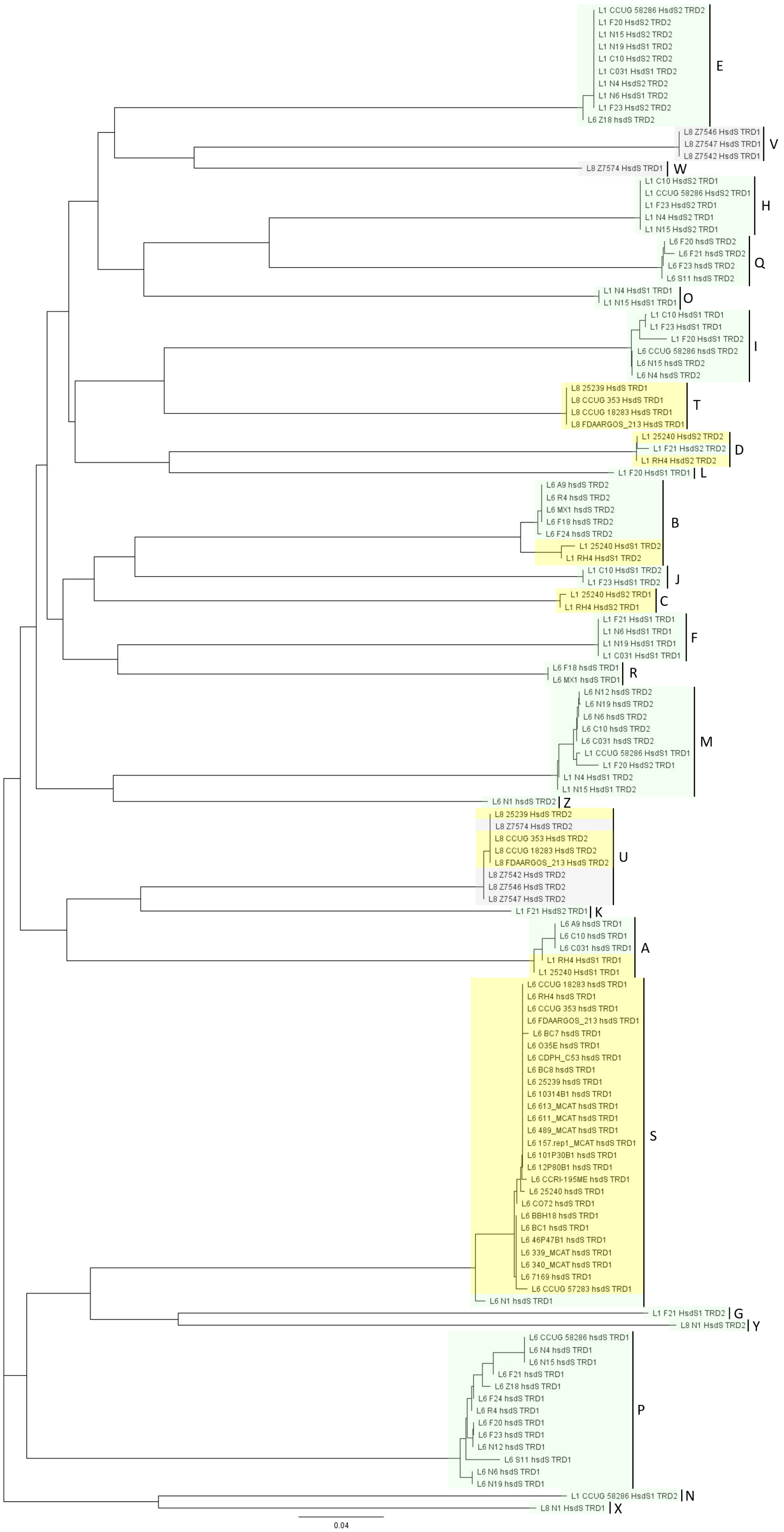
L1 CCUG 58286 HsdS2 TRD2
L1 F20 HsdS2 TRD2
L1 N15 HsdS2 TRD2
L1 N19 HsdS1 TRD2
L1 C10 HsdS2 TRD2
L1 C031 HsdS1 TRD2
L1 N4 HsdS2 TRD2
L1 N6 HsdS1 TRD2
L1 F23 HsdS2 TRD2
L6 Z18 hsdS TRD2
E
L8 Z7546 HsdS TRD1
L8 Z7547 HsdS TRD1
L8 Z7542 HsdS TRD1
V
L8 Z7574 HsdS TRD1
W
L1 C10 HsdS2 TRD1
L1 CCUG 58286 HsdS2 TRD1
L1 F23 HsdS2 TRD1
L1 N4 HsdS2 TRD1
L1 N15 HsdS2 TRD1
H
L6 F20 hsdS TRD2
L6 F21 hsdS TRD2
L6 F23 hsdS TRD2
L6 S11 hsdS TRD2
Q
L1 N4 HsdS1 TRD1
L1 N15 HsdS1 TRD1
O
L1 C10 HsdS1 TRD1
L1 F23 HsdS1 TRD1
L1 F20 HsdS1 TRD2
L6 CCUG 58286 hsdS TRD2
L6 N15 hsdS TRD2
L6 N4 hsdS TRD2
I
L8 25239 HsdS TRD1
L8 CCUG 353 HsdS TRD1
L8 CCUG 18283 HsdS TRD1
L8 FDAARGOS_213 HsdS TRD1
T
L1 25240 HsdS2 TRD2
L1 F21 HsdS2 TRD2
L1 RH4 HsdS2 TRD2
D
L1 F20 HsdS1 TRD1
L
L6 A9 hsdS TRD2
L6 R4 hsdS TRD2
L6 MX1 hsdS TRD2
L6 F18 hsdS TRD2
L6 F24 hsdS TRD2
L1 25240 HsdS1 TRD2
L1 RH4 HsdS1 TRD2
B
L1 C10 HsdS1 TRD2
L1 F23 HsdS1 TRD2
J
L1 25240 HsdS2 TRD1
L1 RH4 HsdS2 TRD1
C
L1 F21 HsdS1 TRD1
L1 N6 HsdS1 TRD1
L1 N19 HsdS1 TRD1
L1 C031 HsdS1 TRD1
F
L6 F18 hsdS TRD1
L6 MX1 hsdS TRD1
R
L6 N12 hsdS TRD2
L6 N19 hsdS TRD2
L6 N6 hsdS TRD2
L6 C10 hsdS TRD2
L6 C031 hsdS TRD2
L1 CCUG 58286 HsdS1 TRD1
L1 F20 HsdS2 TRD1
L1 N4 HsdS1 TRD2
L1 N15 HsdS1 TRD2
M
L6 N1 hsdS TRD2
Z
L8 25239 HsdS TRD2
L8 Z7574 HsdS TRD2
L8 CCUG 353 HsdS TRD2
L8 CCUG 18283 HsdS TRD2
L8 FDAARGOS_213 HsdS TRD2
L8 Z7542 HsdS TRD2
L8 Z7546 HsdS TRD2
L8 Z7547 HsdS TRD2
U
L1 F21 HsdS2 TRD1
K
L6 A9 hsdS TRD1
L6 C10 hsdS TRD1
L6 C031 hsdS TRD1
L1 RH4 HsdS1 TRD1
L1 25240 HsdS1 TRD1
A
L6 CCUG 18283 hsdS TRD1
L6 RH4 hsdS TRD1
L6 CCUG 353 hsdS TRD1
L6 FDAARGOS_213 hsdS TRD1
L6 BC7 hsdS TRD1
L6 O35E hsdS TRD1
L6 CDPH_C53 hsdS TRD1
L6 BC8 hsdS TRD1
L6 25239 hsdS TRD1
L6 10314B1 hsdS TRD1
L6 613_MCAT hsdS TRD1
L6 611_MCAT hsdS TRD1
L6 489_MCAT hsdS TRD1
L6 157.rep1_MCAT hsdS TRD1
L6 101P30B1 hsdS TRD1
L6 12P80B1 hsdS TRD1
L6 CCRI-195ME hsdS TRD1
L6 25240 hsdS TRD1
L6 CO72 hsdS TRD1
L6 BBH18 hsdS TRD1
L6 BC1 hsdS TRD1
L6 46P47B1 hsdS TRD1
L6 339_MCAT hsdS TRD1
L6 340_MCAT hsdS TRD1
L6 7169 hsdS TRD1
L6 CCUG 57283 hsdS TRD1
L6 N1 hsdS TRD1
S
L1 F21 HsdS1 TRD2
G
L8 N1 HsdS TRD2
Y
L6 CCUG 58286 hsdS TRD1
L6 N4 hsdS TRD1
L6 N15 hsdS TRD1
L6 F21 hsdS TRD1
L6 Z18 hsdS TRD1
L6 F24 hsdS TRD1
L6 R4 hsdS TRD1
L6 F20 hsdS TRD1
L6 F23 hsdS TRD1
L6 N12 hsdS TRD1
L6 S11 hsdS TRD1
L6 N6 hsdS TRD1
L6 N19 hsdS TRD1
P
L1 CCUG 58286 HsdS1 TRD2
N
L8 N1 HsdS TRD1
X
0.04

Fig S2.

Figure S2: Phylogenetic dendrogram of Type I R-M system TRDs. Neighbour-joining tree with Tamura-Nei genetic distance model of all 124 specificity subunit TRD sequences from three Type I R-M system loci (L1, L6, L8) in 51 *M. catarrhalis* genome strains. Letters A-Z correspond to TRDs in figure 3c. Coloured boxes indicate which phylogenetic lineage each strain belongs to: yellow = RB1 lineage; green = RB2/3 lineage, and grey = divergent phylogenetic lineages.
